# Effect of anti-CD4 mAb induced by inhibiting B cell disorder on immune reconstruction of HIV-infected immunological non-responders

**DOI:** 10.1186/s10020-025-01286-3

**Published:** 2025-06-20

**Authors:** Yi Ouyang, Kang Wu, Lei Fu, Panpan Yi, Da Cheng, Xiaoyu Fu

**Affiliations:** 1 Department of Infectious Diseases, Xiangya Hospital Central South University, No. 87, Xiangya Road, Changsha City, 410008 Hunan Province China; 2 Xiangya School of Basic Medical Sciences, Central South University, Changsha City, Hunan Province 410013 China

**Keywords:** B cell, Anti-CD4 mAb, Antibody-dependent cellular cytotoxicity, HIV

## Abstract

**Background:**

In persons living with HIV, antiretroviral therapy (ART) reduces HIV RNA in their plasma and increases CD4 + T lymphocytes, thus restoring their immune function and reducing mortality rates.

**Methods:**

The heavy and light chains of B cell receptor (BCR) were amplified, sequenced, analyzed, and determined to be anti-CD4 mAb. The cytotoxicity of NK cells mediated by the anti-CD4 mAb was assessed using CCK-8, flow cytometry, ELISA, and western blotting. Detecting the viability/regulation of CD4 cells involved inhibiting the attachment of autoantibodies against CD4 to crucial receptors and detecting the inhibition of key molecules in B cells to produce anti-CD4 mAb in patients with immune non-responders (INR). Furthermore, through Phage Random Peptide Library Screening, we discovered that the AAPMFHSSVQLP-CD4 peptide has an affinity for the anti-CD4 mAb.

**Results:**

Administering anti-CD4 mAb enhanced NK cytotoxicity. The simultaneous administration of anti-CD4 mAb alongside GST-CD4 alleviated the harmful impacts of anti-CD4 mAb on the CD3 + population in humanized mice, and HIV virus (p24). Individuals diagnosed with INR displayed abnormal B cell activity, particularly with elevated BAFFR expression and increased levels of anti-CD4 mAb. Nevertheless, suppression of BAFFR hindered B cell function and decreased the production of anti-CD4 mAb. In HIV-infected individuals, the dysregulation of B-cells led to the production of anti-CD4 mAb, which in turn facilitated NK cell cytotoxicity and the CD4 + T effect by upregulating the expression of BAFFR.

**Conclusion:**

The dysregulation of B-cells in person living with HIV increased the production of anti-CD4 mAb, which in turn promoted NK cell cytotoxicity and the CD4 + T effect.

**Supplementary Information:**

The online version contains supplementary material available at 10.1186/s10020-025-01286-3.

## Highlights

1) B-cell dysregulation increased anti-CD4 mAb levels.

2) B cells are abnormally active in patients with INR.

3) Knockdown of BAFFR obviously reduced the secretion of anti-CD4 mAb.

**Supplementary Information:**

The online version contains supplementary material available at 10.1186/s10020-025-01286-3.

## Introduction

Human immunodeficiency virus (HIV) infection can cause acquired immunodeficiency syndrome (AIDS). In 2021, the World Health Organization (WHO) approximated that there were approximately 38.4 million global HIV-1 infections, with 15% of individuals unaware of their HIV status. Currently, a significant number of individuals with HIV who are receiving antiretroviral therapy (ART) experience positive treatment results, successfully attaining a viral load that cannot be detected (Menéndez-Arias and Delgado 2022; Mataramvura et al. 2023). However, in clinical settings, there is a group of people infected with HIV, which inhibit HIV-1 plasma viremia by ART, who still unable to perform immune reconstitution and are commonly known as immune non-responders (INRs) (Yang et al. 2020). INRs are people living with HIV (PLHIV) who fail to fully restore CD4 + T-cell counts despite complete viral suppression with ART. PLHIV with CD4 T-cell count < 350 cells/mm^3^ after ≥ 2 years of suppressive ART were defined as INR and were compared to immunological responders (IR) with CD4 T-cell count > 500 cells/mm^3^ (Vos et al. 2024). During our investigation of PLHIV infection, we discovered a notable elevation of anti-CD4 mAb in *person* living with INRs (Luo et al. 2017), indicating that anti-CD4 mAb could potentially be responsible for the occurrence of INR. The underlying mechanism behind inadequate immune reconstitution in *person* living with AIDS remains unknown.

Antibody-dependent cell-mediated cytotoxicity (ADCC) is a phenomenon in immunology that merges the innate and adaptive immune responses. NK cells, as specific immune cells, recognize and eliminate target cells that are coated with antibodies in the process known as ADCC (Ji et al. 2019). Numerous studies have indicated that ADCC plays a crucial role in the management of HIV-1 infection (Yaffe et al. 2021; Luo et al. 2022). Nevertheless, a significant proportion of individuals receiving antiretroviral therapy (ART) can only achieve a moderate increase in CD4 + T cells, despite successfully controlling HIV-1 replication. INRs, classified as such, demonstrate a significantly higher death rate when compared to immunological responders (IRs) (Robbins et al. 2009). In INRs, CD4 monoclonal antibodies (mAbs) have been explored as a prospective approach to bolstering ADCC. In individuals with chronic hepatitis C virus infection, the combination of a CD4 mAb and a therapeutic vaccine resulted in a stronger ADCC response compared to treatment with the vaccine alone (Strunz et al. 2018). Therefore, the use of CD4 mAb shows potential in enhancing the antibody-dependent cellular cytotoxicity immune response in individuals with immune-mediated neutropenia.

Autoantibodies targeting CD4, which selectively attach to the CD4 molecule present on the outer layer of T cells, possess the ability to induce CD4-positive T cell depletion and subsequently hinder immune function (Malle et al. 2023). Autoimmune conditions like HIV, systemic lupus erythematosus (SLE), rheumatoid arthritis (RA), and Sjögren’s syndrome (Li et al. 2022; Chriswell et al. 2022; Yao et al. 2021). have been associated with these autoantibodies. It is worth mentioning that a portion of person with HIV might experience the production of CD4 autoantibodies and active B cells, which could contribute to the advancement of disease activity and severity. Furthermore, it has been demonstrated that anti-CD4 monoclonal antibodies can stimulate the activation of memory B cells in individuals with INRs, resulting in the generation of potent antibodies that effectively neutralize the specific antigen. Moreover, a recent investigation conducted by Nahmad and colleagues confirmed anti-CD4 mAb can be produced by active B cells (Nahmad et al. 2020). Thus, anti-CD4 mAb may be produced by abnormally active B cells in patients with INRs.

In this study, we examined the potential role of anti-CD4 mAb in HIV-infected individuals. In vitro, we explored the effect of anti-CD4 mAb on NK cell activity or apoptosis, specifically focusing on the release of Perforin, Granzymes B, and the expression of TNF-α/TNFR-I. Additionally, we also investigated the function of anti-CD4 mAb in CD4-positive T cells, the apoptosis of CD4-positive T cells, and natural killer cell cytotoxicity. Furthermore, administration of anti-CD4 mAb may reduce the level of HIV virus, and the inhibition of BAFFR resulted in impaired B cell function and diminished production of anti-CD4 monoclonal antibody in INR patients.

## Materials and methods

### Study subjects

Eight healthy controls and 16 HIV-infected ART-treated subjects (8 HIV-ART aviremic subjects and 8 HIV-INR) were studied. HIV-infected subjects had had ART for at least 2 years with undetectable viral loads (plasma HIV RNA < 50 copies/ml). All participants had received the 2018–2019 influenza vaccination (Fluvirin; Glaxo-Smith Kline). The clinical characteristics of the participants are shown in Table 1. Briefly, blood samples were collected on day 0 (D0) before vaccination, days 7 to 10 (D7) postvaccination, and days 14 to 21 (D14) postvaccination. All participants had received the influenza vaccine in the previous year. The Xiangya Hospital Central South University (Ethics number: 202103437) approved this study; all participants provided written informed consent.

**Table 1:** Clinical characteristics on HIV immune response and lack of immune response

Characteristic	Healthy group (*N* = 8)	HIV + Responders (*n* = 8)	HIV + nonresponders (*n* = 8)	*P* value (responders vs. Nonresponders)
Sex (female/male)	5:3	2:6	1:7	>0.99
Age (y)	42 (36–51)	41 (32–51)	43 (35–53)	0.406
CD4 + T cell counts (cells/ul)	742 (524–934)	660 (503–814)	269 (246–296)	<0.001
ART treatment time (y)		4.85 (3.5–6)	6 (3–8)	0.115

### Animal modeling

The Institutional Animal Care and Use Committee (IACUC) granted approval for animal testing conducted at Xiangya Hospital Central South University (Ethics number: 202103437). In this study, experiments were conducted using Gt (ROSA)26Sortml(Luc)Kacl Tg(UBC-CCR5,-CD4)19Mnz/J humanized mice (weight, 20–25 g; age, 8 weeks). These transgenic mice, which harbor the HIV virus, are regulated by the Gt (ROSA)26Sor promoter. They express CD4 and CCR5, making them an ideal model for evaluating clinical drugs and vaccine screening. The mice were obtained from The Jackson Laboratory (Stock No: 023451). To blocking anti-binding polypeptide sites on mouse CD4 cells in humanized mice, we injected the recombinant antibody and positive IgG (5ug/mL, 100ul) by tail vein once every three days. At the 2nd, 4 th, and 6 th weeks after the administration, mouse serum was collected respectively. There are 12–15 mice in each group. Finally, 8 mice were selected for statistical analysis. The mice were housed with 12/12 night/day cycles and 60% relative humidity, temperature was kept at 22 °C. Food and water were freely available to the mice.

### Constructing recombinant plasmids to synthesize anti-CD4 mAb

The gene responsible for encoding the anti-CD4 mAb was successfully inserted into a recombinant plasmid. Subsequently, the recombinant plasmid was introduced into HD CHO-S cells on transfection culture flask (Speed: 150 rpm/min) to facilitate the stable expression and synthesis of the anti-CD4 autoantibody. The transfection dose is 10 mL of cell suspension per inoculation (5.0 × 10^5^/mL) use 1 mL basal DMEM medium diluted with 7 µg plasmid and 21 µg Transfection reagent. The purification of the anti-CD4 mAb from the host cells was achieved through the utilization of protein A affinity chromatography. Design corresponding primers based on the selected monoclonal antibody sequences. The sequences are as follows: CD4-h-H-F: CAGCCGGCCAGGCGCGCEGTACGAAGCTTGGCCCAGCCGGCCCAGGTGCGG, CD4-H-H-R: ATGGGECCTTGGTGGAGGCCGCGGCCG CTGAGGAGACGGTGACCGTGGTECCTTG; CD4-H-L-F: ATGGTGEAGCCACA GTTEGCGCGGCCGCTCGTTTCTGTTCTCTTTCCETTAGGGG, CD4-H-L-R, CA GCCGGCCAGGEGCGCCGTACGAAGCTTGGCCCAGCCGGCCGAAATTGTG.

### Isolation and purification of NK cells and CD4 + T cells

The peripheral blood mononuclear cells (PBMC) were isolated from peripheral blood. The PBMC suspension was split into two 1.5 mL Eppendorf tubes, centrifuged, and the supernatant discarded. Cells were resuspended, with each 80 µL Buffer containing 10^7^ cells. Add 20 µL for every 10^7^ cells, mix NK MicroBeads or CD4 MicroBeads, and incubate at 4–8 °C for 15 min. Wash cells with 1 mL Buffer, centrifuge, discard supernatant, and resuspend in 500 µL Buffer. Place the MS separation column in the magnetic-activated cell sorting (MACS) separator, rinse with 500 µL Buffer, pass 500 µL cell suspension through, and rinse with 500 µL Buffer. Repeat the wash three times, collect the effluent containing non-NK cells or CD4 + T cells, and remove them. Rinse the separation column with 1000 µL Buffer under pressure to collect NK cells or CD4 + T cells.

### NK-cell killing assays (Direct Cytotoxicity and ADCC)

NK cells were co-cultured with anti-CD4 mAb. Briefly, NK cells were seeded at a density of 5 × 10^5^ cells in a 6-well plate and allowed to incubate overnight. Subsequently, the cells were co-cultured with an anti-CD4 mAb (5 µg/mL) for a duration of 24 h. Following this incubation period, the cells were harvested, subjected to staining with anti-CD16-FITC (#ab239254, Abcam, UK) for 30 min, and subsequently analyzed using flow cytometry techniques.

NK Cells and CD4 + T cells co-cultured with anti-CD4 mAb. 50 µL CD4 + T cells (5 × 10^5^/mL) were inoculated into a 96-well V-plate. At the same time, 50 µL NK cells (1.5 × 10^6^/mL) were added. The final ratio of NK: CD4 cells was 3: 1. 5 µg/mL of anti-CD4 mAb protein was added to a 96-well culture plate and cultured at room temperature for 15 min. Then, the 96-well plate was cultured at 37℃ for 6 h, and then it was transferred to 4℃ for refrigeration. The next morning, flow antibody CD3 and CD4 staining was performed. For blocking experiments, target cells were incubated with Fc-blocking antibodies at a concentration of 10 µg/mL for 30 min at 4 °C. Then follow-up related experiments were carried out.

Detection of the degree of CD4 + T cell lysis. After co-culture overnight, supernatants were collected and analyzed using a PerkinElmer Wizard counter (Waltham, MA). CD4 + T cell specific lysis was calculated using the formula: % lysis = 100 x (ER - SR)/(MR - SR), where ER, SR, and MR represent experimental release, spontaneous release, and maximum release, respectively.

### Flow cytometry

The annexin V-FITC/PI apoptosis detection kit (#6592, Cell signaling technology) was used to detect the cell apoptosis according to the manufacturer’s instructions. Following a centrifugation at a force of 1000 g for a duration of 5 min, the cells were suspended again in a binding buffer with a concentration of 1×. To each group, 5 µL of Annexin V-FITC and 7 µL of PI were introduced, and then incubated at room temperature for 15 min. The evaluation of cell apoptosis was conducted by employing flow cytometry (Cytoflex, Beckman, Coulter, CA, USA).

R-phycoerythrin (PE) Conjugation Kit/R-PE Labeling Kit (#ab102918, Abcam, UK) and FITC-labeled anti-CD16 (#ab239254, Abcam, UK) were procured from Abcam. The anti-CD4 mAb was conjugated with PE, while NK cells were labeled using FITC-conjugated anti-CD16. Flow cytometry was employed to evaluate the binding of anti-CD4-PE mAb to human CD4 + T cells. Staining CD4 cells with anti-CD3+ (#ab11089, Abcam) was performed to prevent interference from competing antibody sites of the anti-CD4 mAb, thereby ensuring accurate detection results.

Flow cytometry was used to assess intracellular cytokine (ICC) assays, including IFN-γ and CD107a/b, following the previously described method (Lin et al. 2014). To assess the intracellular expression of IFN-γ cytokine, cells were subjected to pp65 PepMix treatment and aliquots were taken. Afterward, the cells were rinsed and marked with PE–CD8 (#ab239316, Abcam) for a duration of 20 min at a temperature of 4 °C. Afterwards, a 4% permeabilization was carried out with Cytofix/Cytoperm (Pharmingen), and the cells were stained with allophycocyanin (APC)-IFN-γ (#562017, BD Biosciences) for a duration of 30 min. CD107a-FITC (#560949/#561069 BD Biosciences) was introduced to the cells for evaluating the presence of CD107a co-expression. Following incubation with anti-CD4 mAb for a duration of 4 h, the cells were subsequently harvested and assessed using flow cytometry.

PBMCs was separated from whole blood by Ficoll Paque Plus (GE Healthcare) density gradient in Leucosep tube (Greiner Bio). Then, PBMC from different patients were separated by Easysep human B cell enrichment kit (STEMCELL Technologies). CD19+ (#ab320733, Abcam) and CD20+ (#ab64088, Abcam) positive B cells were obtained by flow labeling.

Cells were collected and labeled with Anti-BAFFR antibody (#ab320843, Abcam) at 4℃ for 20 min. Subsequently, anti-CD19 (#ab320733, Abcam) stained the cells for 30 min. Finally, the cells were harvested and evaluated by flow cytometry.

### CCK-8 assay

The assessment of cells was conducted using the Cell Counting Kit-8 (CCK8, Beyotime, Shanghai, China) following the guidelines provided by the manufacturer.The cells were placed in 96-well microplates (Corning, USA) at a concentration of 5 × 10^3^ per well and then exposed to PBS, lgG, and anti-CD4 mAb.After incubating for 24 h, 10 µL of CCK-8 reagent was introduced into each well and left for an extra 2 h.The microplate reader (Bio-Rad, Hercules, CA, USA) was used to measure absorbance readings at 450 nm, with empty wells acting as blanks. The obtained data enabled the assessment of cell growth.The experiments were carried out three times.

### Western blot

The extracted cells and tissues were obtained using RIPA buffer (Beyotime, Shanghai, China) according to previously documented procedures (Ye et al. 2019a, b; Huang et al. 2021). Afterwards, the PVDF membrane was obstructed using 5% BSA in PBST for 1 h. Following that, the membrane was incubated overnight at 4 ℃ with primary antibodies. After three washes, the membrane was treated with secondary antibodies at room temperature for 1 h. Ultimately, the immunoblot was observed utilizing enhanced chemiluminescence (ECL Kit, Advansta) as per the given procedure. The evaluation was made on the strength of the Western groups.

### Quantification of HIV-1 mRNA

Peripheral blood of mice was collected, and mRNA from whole blood samples was extracted using the MagMAX-96 Blood RNA Isolation Kit (AM1837, Thermo Fisher Scientific). Alternatively, RNA was isolated from purified T cells with MagMAX-96 for Microarrays Total RNA Isolation Kit (AM1839, ThermoFisher Scientific). mRNA was then converted to cDNA with the SuperScript VILO cDNA Synthesis Kit (11754250, ThermoFisher Scientific), followed by RT-PCR (qRT-PCR) for HIV-1 pol-1 according to our previous procedure (Li et al. 2020, 2023). HIV-1 pol-1 was amplified with POL-1 forward primer (POL-1-F), 5′-AGCAGGAAGATGGCCAGTAA-3′ and reverse primer (POL-1-R), 5′-GGATTGTAGGGAATGCCAAA-3′. The following cycling parameters were used: 95.0 °C for 2 min followed by 32 cycles at 95.0 °C for 10 s, 55.0 °C for 15 s, and 72.0 °C for 40 s, with plate readi Cycle threshold (Ct) values were calibrated using standard curve generated with the standard samples with known amounts of viral copies.ng performed at 80.0 °C for 2 s and 85.0 °C for 2 s.

### Elisa

The concentrations of Granzymes B and TNF-α in the supernatants of cell cultures were quantified using an ELISA development kit (R&D) in accordance with the manufacturer’s guidelines. The levels of perforin were determined using a human perforin ELISA kit (Invitrogen) following the manufacturer’s instructions.

### Immunofluorescence (IF) staining

The immunostaining procedure was conducted according to previously published methods (Ye et al. 2019a, b). To summarize, cells were first treated with a 4% paraformaldehyde solution for 10 min to fix them, and then permeabilized with a 0.5% Triton X-100 solution for 3 min. Afterwards, the cells were rinsed with TBST three times and subsequently obstructed using a blocking buffer composed of 5% goat serum in 1× PBST for a duration of 1 h. Following this, the cells were exposed to the designated antibodies for an extended period at a temperature of 4 °C and subsequently stained with DAPI (Sigma-Aldrich, St. Louis, MO, USA). The images obtained were taken with a Zeiss (Oberkochen, Germany) fluorescence microscope.

### Phage random peptide library screening

A random peptide phage-displayed library (Ph.D. 12-mer) conducted as previously described (Deng et al. 2021). Anti-CD4 antibodies were immobilized onto a solid support, followed by the addition of a phage display library containing random peptides. The target phages were isolated from the library through iterative cycles of incubation with the antibodies, involving binding, elution, and amplification processes. This approach facilitated the enrichment of specific phages, which were subsequently identified and characterized. Positive phage clones were subjected to gene sequencing analysis to determine their sequences. Ultimately, a polypeptide with high affinity for the anti-CD4 mAb was identified and designated as the CD4 peptide (Supplementary Fig. 1A).

### GST-pull down

GST pull-down assays were conducted as described (Han et al. 2019). The gene sequence of the CD4 polypeptide, as determined in the aforementioned experiment, was inserted into the pET-GST vector to construct the pET-GST-CD4 peptide plasmid. This plasmid was then transfected into Escherichia coli, followed by extraction and purification after amplification. An assay sub-reaction system was established, wherein the recombinant anti-CD4 mAb and the GST-CD4 peptide were added and incubated. Post-incubation, the GST-Pull Down method was employed to isolate and purify the “anti-CD4 mAb-GST-CD4 peptide” binding complex. The material was subjected to SDS-PAGE and subsequently transferred onto a membrane. The membrane was incubated with an HRP-labeled anti-human IgG secondary antibody, and an ECL reagent was used to detect the presence of a luminescent band. This procedure was conducted to further elucidate the binding interaction between the anti-CD4 mAb and the CD4 polypeptide (target antigen).

### RNA interference

The application of RNA interference was conducted utilizing the short hairpin RNA (shRNA) technology (Ye et al. 2019a, b). The methodology for constructing the shRNA expressing H1 retroviral system has been previously elucidated (Ye et al. 2019a, b; Wu et al. 2020). In order to target human BAFFR, shRNA-mediated interference was carried out in human cells employing a pSUPER.Retro.puro vector (OligoEngine, Seattle, WA, USA) that encodes the specific shRNA sequence. The BAFFR target sequences was 5′-UUCCAUGCUCUCCAUCUUCdTsdT-3′ and scrambled (sh-NC) with the antisense strand 5′-GACUACUCGUUGAUGGACUdTdT-3′.

### Statistical analysis

SPSS 19.0 (IBM, Manassas, VA, USA) was utilized for all statistical analyses, while GraphPad Prism 5.0 (GraphPad Software, La Jolla, CA, USA) was employed to generate plots. The Scheffe test was used to determine statistically significant differences among more than two groups through oneway analysis of variance (ANOVA). At least three independent trials were performed, with each experiment being conducted in triplicate.

## Results

### Recombinant and amplified anti-CD4 mAb

**Fig. 1:**
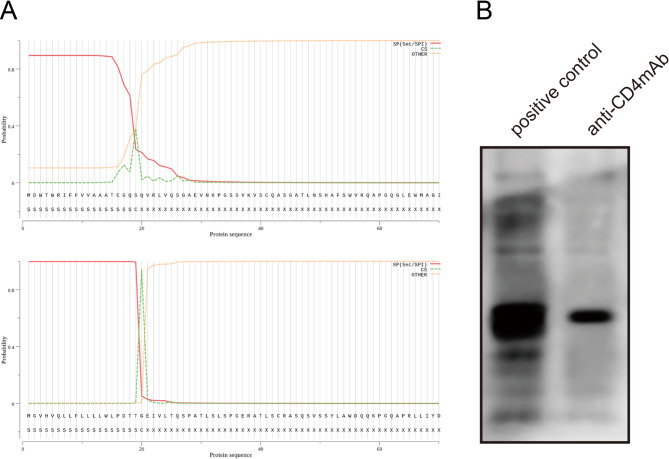
Recombinant and amplified anti-CD4 mAb. **A** B cells with the highest titer of anti-CD4 IgG, amplified the heavy and light chains of BCR, were sequenced and identified as monoclonal antibodies. **B** Detection of recombinant protein against CD4 autoantibody by Western blot

To produce the recombinant and amplified anti-CD4 mAb, the CD4 gene was cloned into a plasmid vector that is compatible with expression in Chinese hamster ovary (CHO) cells. Subsequently, the aforementioned plasmid was transfected into CHO cells, enabling the expression and subsequent purification of the anti-CD4 mAb from the CHO culture. We found that B cells exhibiting the most elevated concentration of anti-CD4 IgG, underwent amplification of both heavy and light chains of BCR, and subsequently underwent sequencing and identification as anti-CD4 mAb (Fig. 1A). The heavy chain sequence exhibits a significant homology of 90.28% with the established anti-cardiolipin autoantibody L22582 (39), and it corresponds to the IGHV1-69*01 F strain. The CDR3 length of this sequence comprises 27 amino acids and encompasses 30 mutations in the V region, resulting in a mutation rate of 18.5% (30/162). On the other hand, the light chain demonstrates a high identity of 98.2% with the recognized anti-human CD40 antibody (40), and it originates from the germline of IGKV3D-11*02 F. The CDR3 length of the light chain consists of 8 amino acids. To test whether the anti-CD4 mAb was a recombinant protein, we performed western blot assay found that anti-CD4 mAb was a recombinant protein (Fig. 1B).

### Anti-CD4 mAb mediate NK cell cytotoxicity in vitro

**Fig. 2:**
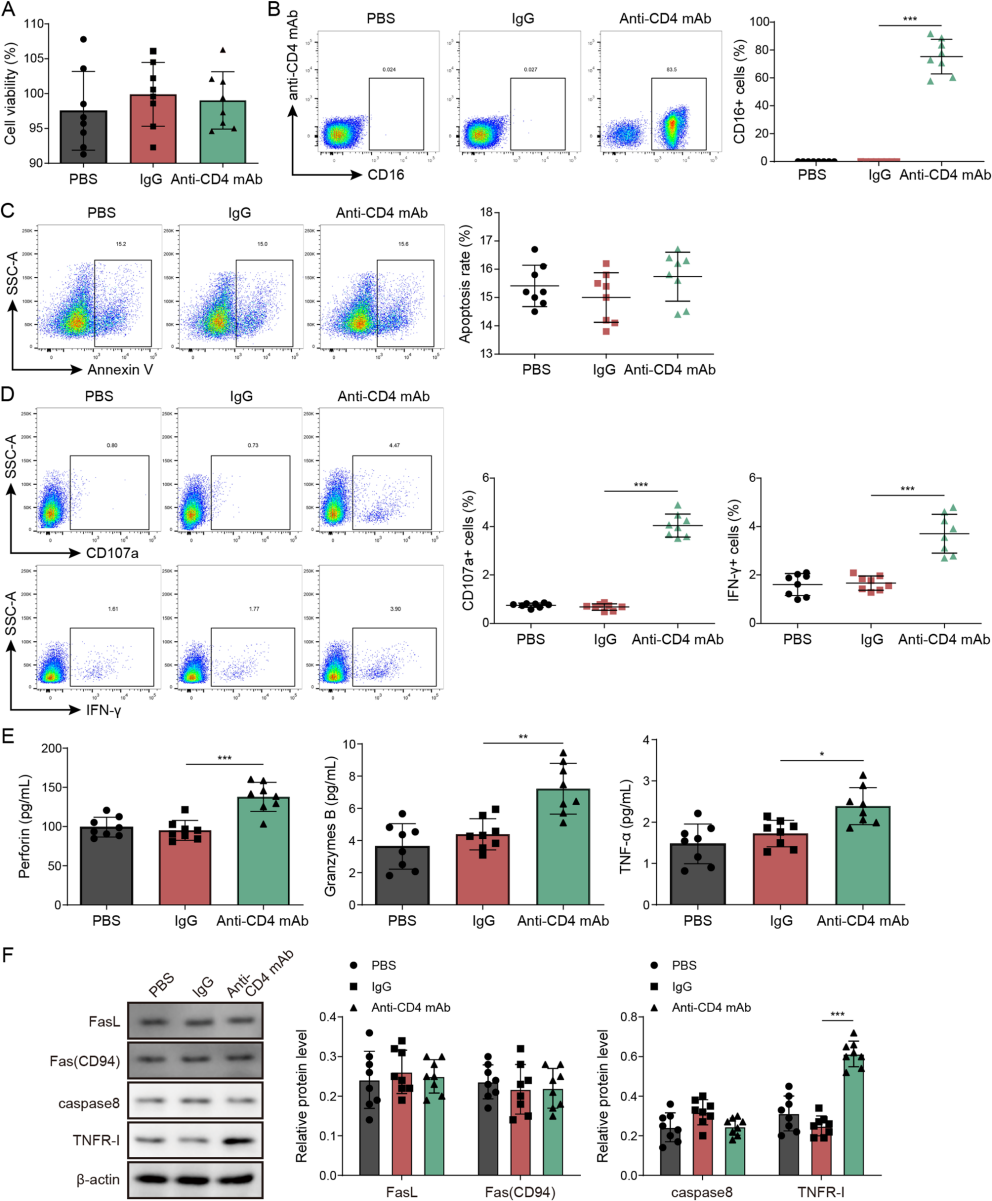
Anti-CD4 autoantibodies mediate NK cell cytotoxicity in vitro. **A** The cell viability was detected by CCK-8. **B** Anti-CD4 mAb -PE labeled anti-CD4 mAb and antibody CD16-FITC labeled NK cells. The binding relationship between NK cells and anti-CD4 mAb was detected by flow cytometry. **C** NK cell apoptosis ratio was detected by flow cytometry. **D** The expression of cytokine γ-interferon (IFN-γ) and CD107a was detected by flow cytometry. **E** The levels of Perforin, Granzymes B, TNF-α in cell supernatant was detected by ELISA. **F** The expressions of FasL, Fas (CD94), caspase8 and TNFR-I in NK cells were detected by Western blot. Data are represented as mean ± SD of 8 separate experiments. ^*^P < 0.05, ^**^P < 0.01, and ^***^P < 0.001 vs. the lgG cohort

We subsequently examined the impact of anti-CD4 mAb on the cytotoxicity and apoptosis of NK cells. Our findings indicated that the presence of anti-CD4 autoantibodies did not alter NK cell activity, as determined by CCK8 analysis, when compared to NK cells treated with PBS or IgG (Fig. 2A). Furthermore, flow cytometry analysis revealed that NK cells bind to anti-CD4 mAb (Fig. 2B) and anti-CD4 mAb did not induce apoptosis in anti-CD4 autoantibodies-treated NK cells, relative to PBS and IgG-treated NK cells (Fig. 2C). However, it was observed that the use of anti-CD4 mAb significantly augmented the cytotoxicity of NK cells (Fig. 2D). Subsequently, our investigation revealed that the enhanced NK cell cytotoxicity mediated by anti-CD4 mAb was associated with the secretion of Perforin, Granzymes B, and the expression of TNF-α/TNFR-I, while no significant correlation was found with FasL, Fas(CD94), and caspase 8 (Fig. 2E and F). These findings suggest that anti-CD4 mAb can facilitate NK cell cytotoxicity by promoting the secretion of Perforin, Granzymes B, and TNF-α/TNFR-I.

### Effect of ADCC mediated by anti-CD4 mAb on CD4 + T cells

**Fig. 3:**
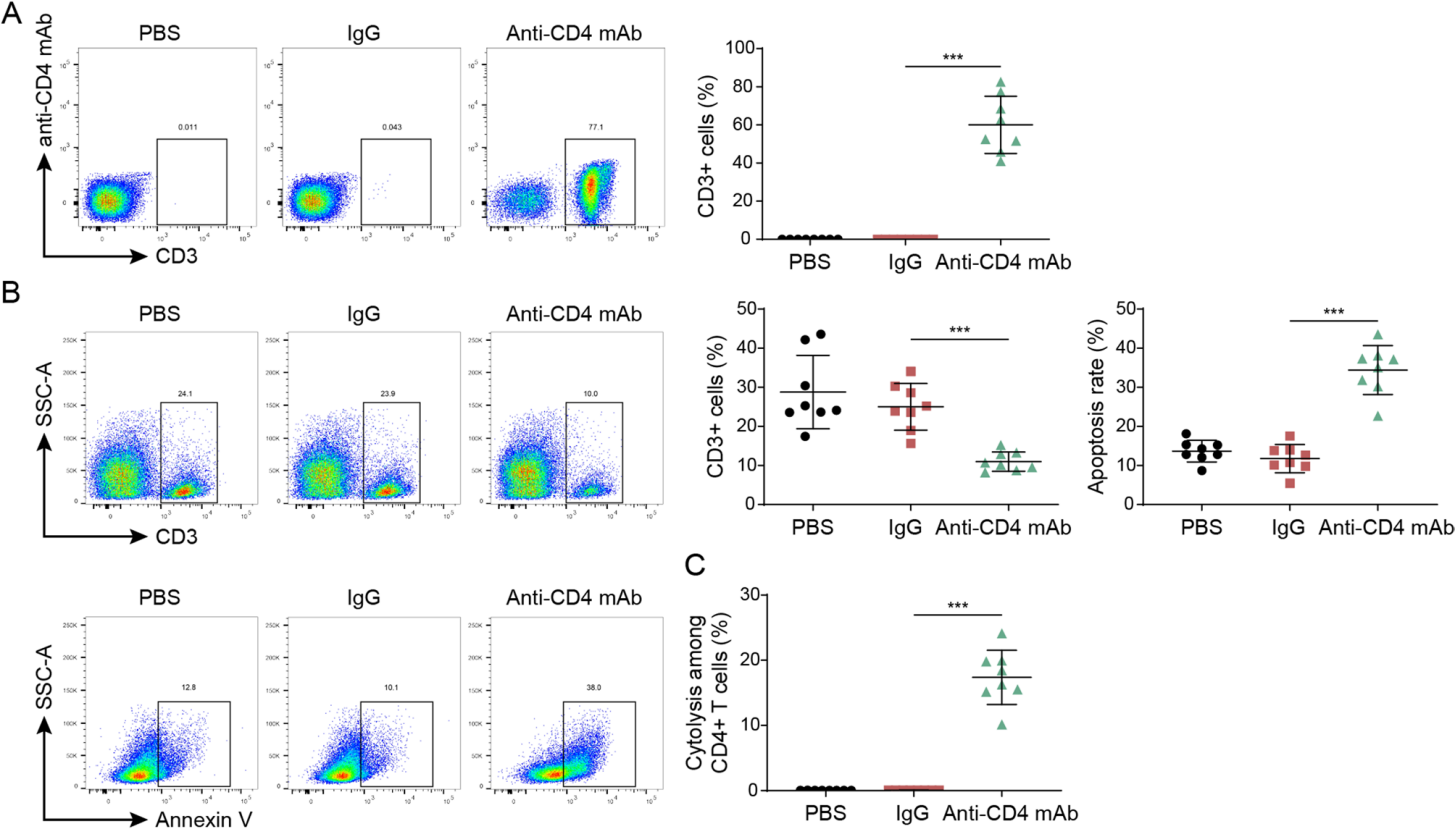
Effect of ADCC mediated by anti-CD4 mAb on CD4 + T cells. **A** Fluorescent labeling (anti-CD4 mAb-PE labeling) was performed on the prepared anti-CD4 mab. The binding of anti-CD4 mAb to CD4 + T cells (CD3-FITC labeling) was detected by flow cytometry. **B** The number and apoptosis ratio of CD4 + T cells were detected by flow staining. **C** The degree of CD4 + T cell lysis was detected. Data are represented as mean ± SD of 8 separate experiments. ^***^P < 0.001 vs. the lgG cohort

The relationship between increased surface CD4 expression on CD4 + T cells and T-cell activation has been established (Hunt et al. 2003). In order to investigate the potential of anti-CD4 mAb on ADCC, a culture was set up with NK cells (peripheral blood of healthy volunteers) and CD4 + T cells (peripheral blood of AIDS patients), using IgG or PBS as controls. Anti-CD4 mAb were stained with PE-conjugated anti-CD4 mAb. Notably, flow cytometry analysis demonstrated that anti-CD4 mAb can bind to the CD4 + T cells in vitro (Fig. 3A) and anti-CD4 mAb reduces CD4 cell activity and increases CD4 cell apoptosis (Fig. 3B). Furthermore, we also detected CD4 + T cell lysis, and found that anti-CD4 mAb can target and kill CD4 + T cells through ADCC effect (Fig. 3C). In addition, we also investigated the effects of anti-CD4 mAb-mediated ADCC on CD4 + T cells, and found that Fc-blocking antibodies inhibit the killing effect of anti-CD4 mA on CD4 + T cells by flow cytometry on CD4 cell activity and increases CD4 cell apoptosis (Supplementary Fig. 4A). Fc-blocking antibodies also inhibit the CD4 + T cell lysis (Supplementary Fig. 4B). Therefore, it can be concluded that in vitro, anti-CD4 mAb has the ability to inhibit CD4 + T cell activation by NK cells.

### Anti-CD4 mAb interacts with AAPMFSHSVQLP-CD4

**Fig. 4:**
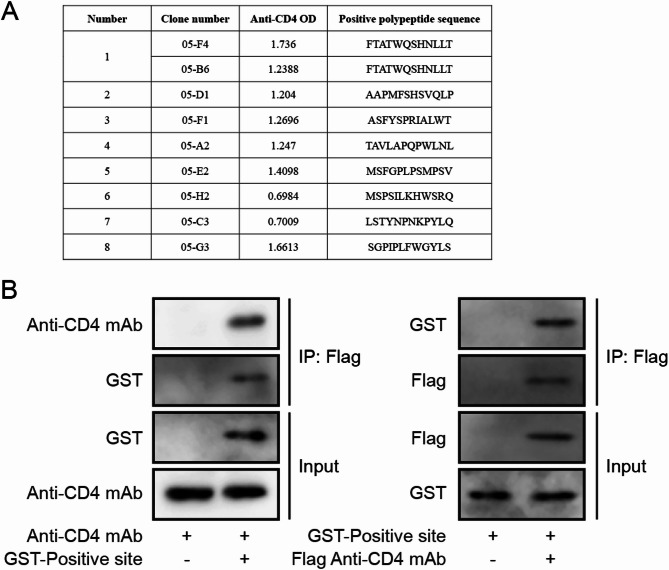
Detection of Anti-CD4 mAb Binding to Target Epitope. According to the previous results, the current candidate peptides: (1) FTATWQSHNLLT; (2) AAPMFSHSVQLP; (3) MSFGPLPYMPSV; (4) LVKPPTYIAHLP; (5) LTRSPTIANHHY; (6) LTAPYVIAHQLN; (7) MSYHWTVNHIPA; (8) FVPYTSVKFSSR. Select AAPMFHSSVQLP, referred to as Positive site. **A** GST-Pull Down Detection of Anti-CD4 mAb Binding to CD4 Peptide. Data are represented as mean ± SD of 8 separate experiments

To identify potential peptide sequences, we further studied the peptide results from Phage Random Peptide Library Screening. Of note, we found 8 peptide sequences, including FTATWQSHNLLT, AAPMFSHSVQLP, MSFGPLPYMPSV, LVKPPTYIAHLP, LTRSPTIANHHY, LTAPYVIAHQL, MSYHWTVNHIPA, and FVPYTSVKFSSR (Fig. 4A). In order to determine the specific region of anti-CD4 mAb that interacts with AAPMFSHSVQLP-CD4, we generated anti-CD4 mAb and Flag-anti-CD4 mAb for GST-pulldown assays. The results demonstrated that AAPMFSHSVQLP-CD4 directly binds to anti-CD4 mAb (Fig. 4B), while no significant direct binding was observed between anti-CD4 mAb and the other seven CD4 polypeptides (Supplementary Fig. 1B). Therefore, we can conclude that AAPMFSHSVQLP-CD4 interacts with the anti-CD4 mAb.

### Effects of blocking the binding of anti-CD4 mAb to crucial targets on the survival/normalization of CD4 cells

**Fig. 5:**
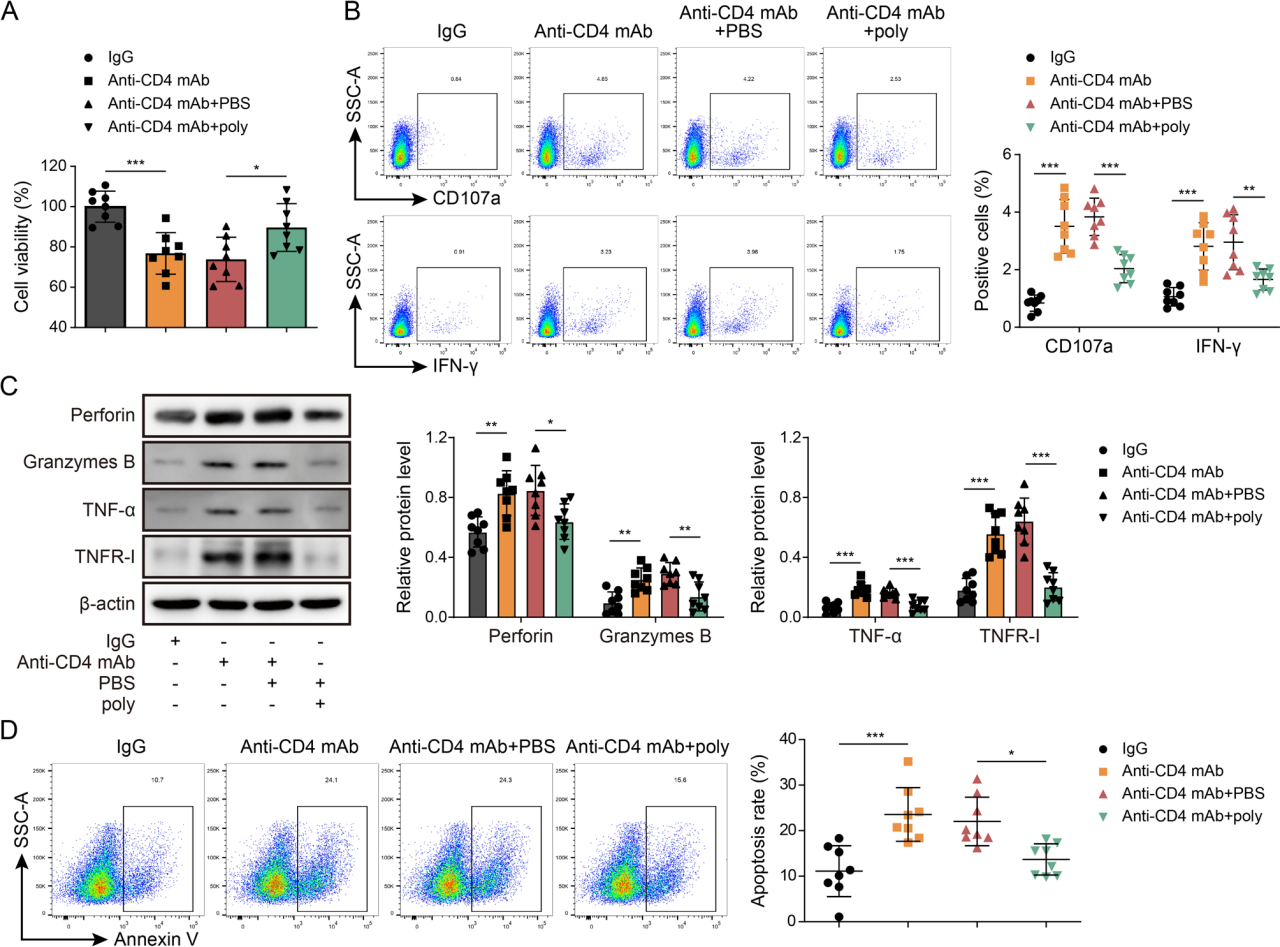
Effects of blocking the binding of anti-CD4 mAb to key targets on the survival/normalization of CD4 cells. **A** The CD4 + T cell activity was detected by CCK-8. **B** The levels of NK cytokine γ-interferon (IFN-γ) and CD107a was detected by flow cytometry. **C** Detection of Perforin, Granzymes B, TNF-α and TNFR-I in cells by Western blot. **D** Flow cytometric double staining was used to detect the apoptosis level of CD4 + T cells. Data are represented as mean ± SD of 8 separate experiments. ^*^P < 0.05, ^**^P < 0.01, and ^***^P < 0.001 vs. the lgG or anti-CD4 mAb + PBS cohort

In order to investigate the impact of anti-CD4 mAb binding to key targets on the survival and normalization of CD4 cells, we conducted co-cultures with NK cells and CD4 + T cells. These cells were divided into four groups: IgG, anti-CD4 mAb, anti-CD4 mAb + PBS, and anti-CD4 mAb + CD4 polypeptide (Fig. 5A-D). Our findings indicate that the restoration of anti-CD4 mAb’s effect on CD4 + T cell activity by CCK8 assay (Fig. 5A), NK cytotoxicity by flow Cytometry (Fig. 5B), the secretion of Perforin and Granzymes B and the expression of TNF-α/TNFR-I by western blot (Fig. 5C), and CD4 + T cell apoptosis by flow Cytometry (Fig. 5D) can be achieved by blocking the binding peptide (and anti-CD4 mAb + CD4 polypeptide). Hence, the data presented herein indicate that inhibiting the interaction between anti-CD4 mAb and CD4 + T cells could potentially enhance various aspects of CD4 + T cell functionality, including NK cytotoxicity, the secretion of Perforin and Granzymes B, the expression of TNF-α/TNFR-I, and the regulation of CD4 + T cell apoptosis.

### Effects of blocking anti-CD4 mAb treatment on mouse CD4 cells in humanized mice

**Fig. 6:**
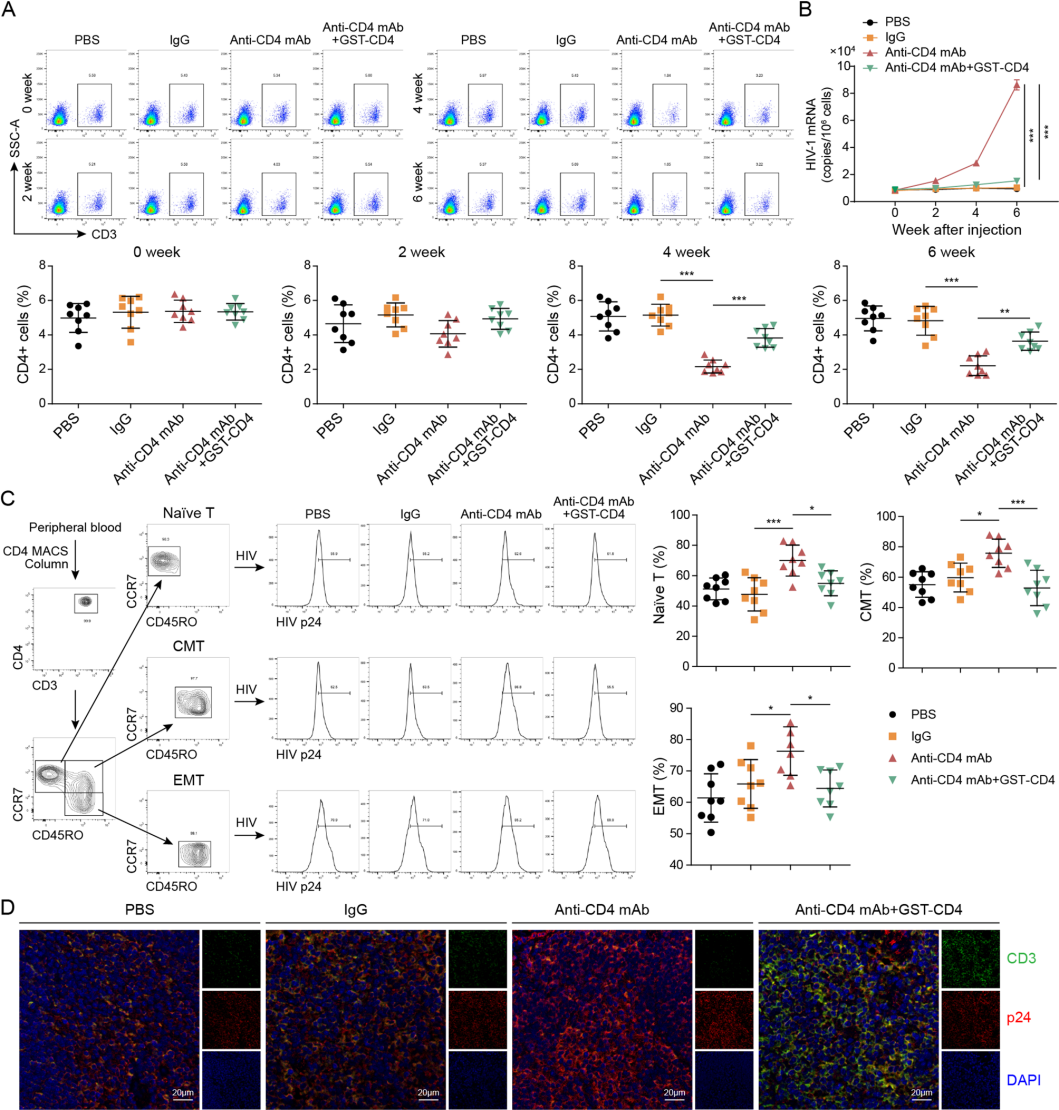
Effects of blocking anti-binding polypeptide sites on mouse CD4 cells in humanized mice. The mouse was divided into four groups: PBS, positive IgG, anti-CD4 mAb, anti-CD4 mAb + GST-CD4 polypeptide group. The tail vein injection technique is employed to administer injections at a frequency of once every three days, with a final concentration of 5ug/ml for both recombinant antibody and positive IgG. Each mouse receives an injection volume of 100 µL. The mice are collected prior to drug administration and at the 2nd, 4 th, and 6 th week following drug administration. **A** The number of CD4 + T cells in blood was detected by Flow Cytometry. **B** The HIV RNA expression was detected by qRT-PCR. **C** After 6 weeks, peripheral monocytes were isolated by flow cytometry to detect initial CD4 + T (CD3 + CD4 + CD8-CD45RA + CD27+), effector CD4 + T (EMT) (CD3 + CD4 + CD45RA-CD45RO + CCR7–) and memory HIV viral load in CD4 + T (CMT) (CD3 + CD4 + CD45RA–CD45RO + CR7+). **D** The CD4 + T Cells in Mouse Spleen Tissue was detected by Immunofluorescence. Data are represented as mean ± SD of 8 separate experiments. ^*^P < 0.05, ^**^P < 0.01, and ^***^P < 0.001 vs. the lgG or anti-CD4 mAb cohort

A randomized division of humanized mice into four groups was carried out to examine the impacts of anti-CD4 mAb therapy on subsequent alterations. The groups were administered either a control vehicle (PBS and lgG) or various drugs (anti-CD4 mAb and anti-CD4 mAb + GST-CD4) as specified. The humanized mice received recombinant antibody injections via tail vein every three days, with a concentration of positive IgG set at 5ug/ml and a volume of 100ul per mouse. Mouse serum samples were collected prior to treatment and at 2-, 4-, and 6-weeks post-treatment, spanning three consecutive days starting from 3 days before and continuing until 14 days after bacillus Calmette–Guérin inoculation. In Fig. 6A, the administration of anti-CD4 mAb + GST-CD4 resulted in a reversal of the impact of anti-CD4 mAb on the population of CD4 + T cells in the bloodstream of mice, with the most significant effect observed during the fourth week. Furthermore, the application of anti-CD4 mAb + GST-CD4 led to a significant reduction in HIV viral load, as determined by qRT-PCR analysis (Fig. 6B). Additionally, anti-CD4 mAb + GST-CD4 exhibited a notable reversal of the expression of naive CD4 + T cells, epithelial-to-mesenchymal transition (EMT), and cancer-associated fibroblast (CAF) markers in mice treated with anti-CD4 mAb (Fig. 6C). Moreover, the co-administration of anti-CD4 monoclonal antibody (mAb) with GST-CD4 successfully mitigated the deleterious effects of anti-CD4 mAb on the population of CD4 + T cells (CD3+) in the spleen and lymph nodes of humanized mice, as well as the presence of HIV virus (p24) (Fig. 6D). Consequently, the combined treatment of anti-CD4 mAb + GST-CD4 exhibited a significant reversal of the impact of anti-CD4 mAb on CD4 cells in the mouse model of humanized mice.

### Alterations in the functioning of B cells among individuals with HIV infection

**Fig. 7:**
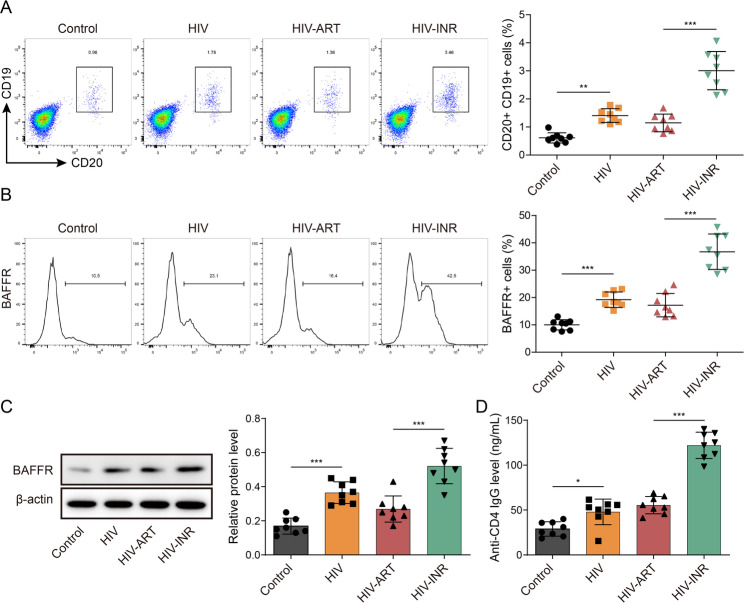
Changes of B cell activity in clinical HIV patients. The patients were divided into four groups: health individuals, HIV-infected patients, ART patients, and INR. **A** Flow cytometric detection and isolation of B cells (CD19, CD20). **B** Flow cytometric detection of BAFFR, BCR, TLR and co-stimulatory molecules. **C** Western blot detected the protein expression of BAFFR, BCR, TLR and co-stimulatory molecules. **D** The content of anti-CD4 mAb was measured by ELISA. Data are represented as mean ± SD of 8 separate experiments. ^*^P < 0.05, ^**^P < 0.01, and ^***^P < 0.001 vs. the control or HIV-ART cohort

In order to assess the alterations in B cell (CD19 and CD20) function among individuals with HIV, our investigation revealed that B cells exhibited abnormal levels of activity in individuals with INR (Fig. 7A). However, no significant disparity in B cell activity changes was observed between HIV-infected participants receiving antiretroviral therapy (ART) and healthy individuals (Fig. 7A). Additionally, we observed a notable increase in the expression of B-cell activating factor receptor (BAFFR) in INR individuals (Fig. 7B and C), while no differences were noted in the expressions of B cell receptor (BCR), Toll-like receptor (TLR), and costimulatory molecules (including CD40, CD80, and CD86) between HIV-infected participants on ART and health individuals (Supplementary Fig. 3). Recent research has indicated that the utilization of anti-CD4 monoclonal antibodies (mAb) has been observed in the management of autoimmune disorders (Xu et al. 2022; Swanstrom et al. 2021). This finding implies that anti-CD4 mAb could potentially be employed as a therapeutic approach for HIV treatment. Furthermore, it was observed that the levels of anti-CD4 mAb were elevated in individuals with INR (Fig. 7D). Consequently, it can be inferred that B cells exhibit abnormal levels of activity in persondiagnosed with INR.

### The effect of knockdown BAFFR on the production of anti-CD4 mAb in B cells of INR individuals

**Fig. 8:**
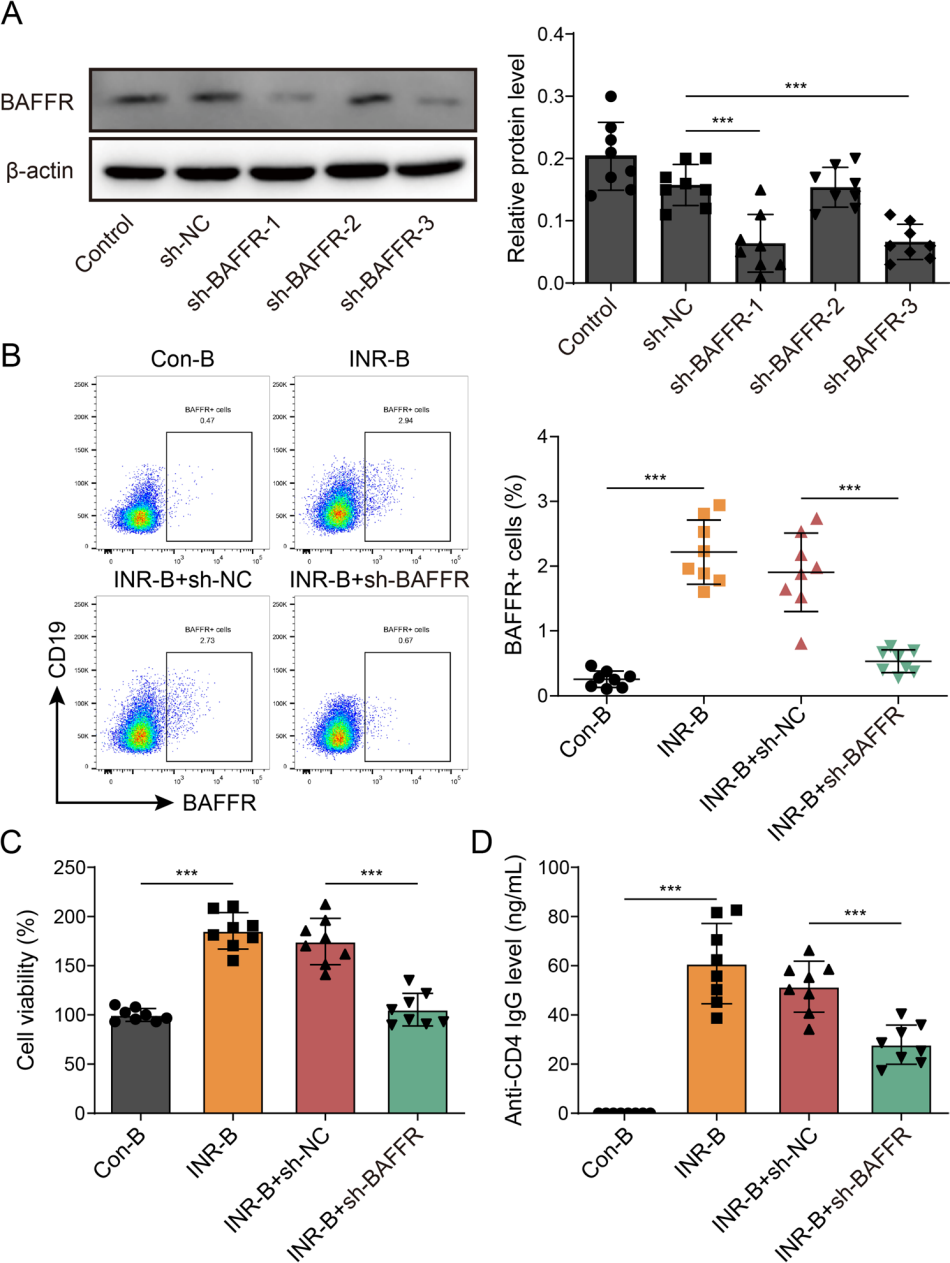
The effect of blocking the key molecules of B cells on the production of anti-CD4 mAb in B cells of patients with INR. The B cells were identified and isolated from healthy and INR patients, and knockdown of BAFFR by lentivirus in these cells. **A** The knockdown efficiency of BAFFR was detected by Western blot. **B** Detection of BAFFR content by Flow cytometry. **C** Detection B cell viability by CCK-8. **D** Detection of anti-CD4 mAb content by ELISA. Data are represented as mean ± SD of 8 separate experiments. ^***^P < 0.001

B-cell-stimulating factor receptor (BAFFR), a significant receptor implicated in the viability of B cells, plays a crucial role (Smulski and Eibel 2018). In order to investigate the impact of BAFFR on the production of anti-CD4 monoclonal antibodies (mAb) in B cells of individuals with INR, we employed shRNA to knock down BAFFR and assess changes in protein levels. As depicted in Fig. 8A, the knockdown of BAFFR in B cells resulted in a respective inhibition of protein expression by 50%, 100%, and 50%, suggesting that knockdown efficiency of BAFFR in protein levels. For subsequent experiments, sh-BAFFR-1 was selected. Additionally, a notable suppression of BAFFR expression was observed in B cells of INR patients following BAFFR knockdown (Fig. 8B). This was accompanied by decreased B cell viability (Fig. 8C) and reduced secretion of anti-CD4 mAb (Fig. 8D). Consequently, the inhibition of BAFFR through knockdown resulted in impaired B cell function and diminished production of anti-CD4 monoclonal antibody in INR patients.

## Discussion

Anti-CD4 antibodies have been investigated as a type of therapeutic agent that target the CD4 receptor on immune cells and a potential treatment for HIV infection (Luo et al. 2017; Laopajon et al. 2023). In the current study, we found that B cell dysregulation induces anti-CD4 autoantibody (IgG type) production to regulate antibody-dependent cell-mediated cytotoxicity and thus affect immune unresponsiveness in HIV-infected individuals. Our data also indicated that blocking the binding of anti-CD4 autoantibodies to key targets could potentially enhance various aspects of CD4 + T cell functionality, including NK cytotoxicity, the secretion of Perforin and Granzymes B, the expression of TNF-α/TNFR-I, and the regulation of CD4 + T cell apoptosis.

In our study, we observed that anti-CD4 antibodies did not induce apoptosis in CD4 cells. In the absence of apoptosis, the potential mechanisms underlying INR predominantly involve hyperactivation of the immune system and dysregulation of the inflammatory response (Xiao et al. 2022; Zhao et al. 2021). Following the initiation of ART in individuals infected with HIV, the immune system progressively recovers, resulting in an increase in CD4 + T cell counts. This rapid immune recovery may precipitate antigen-specific T cell expansion and a cytokine storm. The incidence of INR is intricately linked to an imbalance in the inflammatory response. Specific mechanisms implicated include the dysregulation of inflammatory mediators and immune cell infiltration. In the absence of apoptosis, the potential mechanisms contributing to INR encompass excessive immune system activation, inflammatory response dysregulation, exposure to pathogen antigens, defects in immune regulatory processes, alterations in the tissue microenvironment, enhancement of pathogen-specific immune responses, and the influence of genetic and immunological backgrounds. These mechanisms collectively contribute to the onset and progression of INR. Future research should further explore these mechanisms to provide new ideas for the prevention and treatment of INR.

Recent studies have shown that the ADCC mechanism can be beneficial in the context of HIV infection (Mielke et al. 2021; Forthal and Finzi 2018). By using anti-CD4 antibodies that trigger ADCC, infected CD4-positive T cells can be targeted and eliminated by the immune system, helping to control the spread of the virus. It’s important to note that while ADCC mediated by anti-CD4 antibodies has shown potential in preclinical and early clinical studies, however, there have no approved anti-CD4 antibody therapies specifically for ADCC in the treatment of HIV. Existing data show that ADCC is not only effective in anti-tumor (Modica et al. 2016), and also play an important role in anti-HIV infection (Williams et al. 2015). During our investigation, we discovered that the use of anti-CD4 monoclonal antibody suppressed the functioning of CD4 + T cells and formed a bond with the NK cells.Nevertheless, it is crucial to acknowledge that although anti-CD4 monoclonal antibodies mainly focus on CD4 + T lymphocytes, they can also attach to a specific group of natural killer cells that exhibit CD4 on their exterior.Natural killer cells are a component of the innate immune system and have a function in identifying and eradicating infected cells. The binding of anti-CD4 mAbs to CD4 on NK cells can potentially modulate their activity as well, although NK cells primarily rely on other receptors and mechanisms for their cytotoxic functions.The specific effects of anti-CD4 mAbs on NK cells would depend on various factors, including the antibody’s binding affinity, the density of CD4 expression on NK cells, and the functional consequences of CD4 engagement on NK cell activity. Additional investigation is required to completely clarify the influence of anti-CD4 monoclonal antibodies on the functionality and behavior of NK cells.

Even in individuals who are in good health, there are small amounts of autoantibodies, although the majority of these antibodies have weak binding and do not cause any disease (Siloşi et al. 2016). During the acute phase of HIV infection, there is already the presence of polyclonal B-cell activation and increased levels of autoantibodies, which continue to persist throughout the chronic infection (Shirai et al. 1992; Lane et al. 1983). However, in our study, knockdown of BAFF inhibited the activation of B-cell, and reduced the anti-CD4 mAb. We found that B cells are abnormally active and significantly increased the BAFFR expression and levels of anti-CD4 mAb in INR individuals. The majority, although not all, of this activation is reversed following ART (Nilssen et al. 2004). Multiple research studies have indicated inverse relationships between plasma or serum autoantibodies targeting CD4 + T cells and the count of CD4 + T cells or the progression of the disease (Luo et al. 2017; Müller et al. 1993). Nevertheless, instead of focusing on antibodies specific to the CD4 protein, these studies examined total antibodies that target various surface proteins of CD4 + T cells. Furthermore, the presence of anti-CD4 antibodies was detected in individuals with HIV infection during the early 1990 s (Chams et al. 1988; Martin et al. 1994; Callahan et al. 1992). Nevertheless, the aforementioned studies failed to establish any involvement of anti-CD4 antibody in the development of HIV. It is worth mentioning that individuals who were HIV-positive during the 1990 s were probably not receiving treatment and had a high viral load. On the other hand, participants who did not respond to the current study are not experiencing viral replication and are currently undergoing antiretroviral therapy. However, their CD4 + T-cell counts remain below 350 cells/µL. Therefore, the variations in individuals’ condition could account for the contrasting findings on anti-CD4 IgG between the studies conducted during the pre-ART era (Chams et al. 1991; Sekigawa et al. 1991) and the current study. Moreover, the commencement of ART in people with HIV may result in the advancement of autoimmune disorders, suggesting that the generation of autoantibodies and the emergence of autoimmune ailments could be facilitated by the immune reconstitution triggered by ART (Sheikh et al. 2014; Zandman-Goddard and Shoenfeld 2002). Certainly, our research offers conclusive empirical proof that anti-CD4 possesses the necessary characteristics to impede the activity of CD4 + T-cells through ADCC.

Targeting BAFFR impairs protective antibody responses against other pathogens, including influenza virus, hepatitis B virus, borrelia hermsii infection, and parasites and fungi (Dickinson et al. 2014; Bagheri Yazdi et al. 2022; Khlaiphuengsin et al. 2020; Sakai and Akkoyunlu 2017), which increased the risk of infection. In addition, targeting BAFFR may cause unintended immunosuppressive effects, including reduced B cell numbers, decreased antibody levels, impaired immune memory, and indirect effects on T cell function (Roth et al. 2016; Evans et al. 2023; Towriss et al. 2023; Mackay and Leung 2006). To reduce this negative impact, strategies such as selective targeting, combination therapy, and individualized therapy may be considered.

Inhibition of BAFFR reduces anti-CD4 levels and may lead to immune reconstitution in patients with INR. To further verify the impact of BAFFR inhibition on immune reconstitution in INR patients, experiments in humanized mouse models (such as NSG mouse) and non-human primate models (such as macaques) can be considered (Lu et al. 2023; Singh et al. 2024). Humanized mice have human immune systems and can simulate HIV infection and immune reconstitution processes. The immune systems of non-human primates are highly similar to humans and can better simulate the pathological processes of HIV infection and INR. Thus, inhibiting BAFFR may promote immune reconstitution in INR patients by reducing anti-CD4 antibody levels. Experiments in humanized mice and non-human primate models are feasible ways to test this hypothesis. Humanized mice are suitable for preliminary screening and mechanistic studies, while non-human primates are more suitable for in-depth preclinical studies. Future studies should combine multiple models and multi-omics data to comprehensively evaluate the impact of inhibiting BAFFR on immune reconstitution in INR patients to provide a theoretical basis for clinical treatment.To summarize, our findings indicate that anti-CD4 mAb might be involved in a previously unknown process that hinders the activation of CD4 + T cells by NK cells in individuals with HIV infection. Furthermore, we present supporting evidence of its existence in vivo. The results of our study provide insights into potential novel approaches to suppress the expression of BAFFR, consequently inhibiting the function of B cells and reducing the levels of anti-CD4 monoclonal antibodies following INR.

## Supplementary Information

Supplementary Figure 1. Detection of binding of CD4 autoantibodies to target epitopes. There were 7 polypeptides: 1) FTATWQSHNLLT; 2) MSFGPLPYMPSV; 3) LVKPPTYIAHLP; 4) LTRSPTIANHHY; 5) LTAPYVIAHQLN; 6) MSYHWTVNHIPA; 7) FVPYTSVKFSSR. A. Phage Random Peptide Library Screening. B. GST-Pulldown detection of anti-CD4 mAb binding to CD4 Peptide.

Supplementary Figure 2. Effects of anti-CD4 autoantibodies on CD4+ cells in liver, lung, and lymph nodes in a humanized mouse model. A. Immunofluorescence detection of CD4+ T cells in mouse liver, lung, and lymph node tissues.

Supplementary Figure 3. Changes in B cell activity in clinical HIV patients. The patients were divided into four groups: health individuals, HIV-infected patients, ART patients, and INR. A. Flow cytometric detection of BCR, TLR and co-stimulatory molecules (including CD40, CD80 and CD86). B. Western blot detection of BCR, TLR and co-stimulatory molecules (including CD40, CD80 and CD86). Data are represented as mean ± SD of 8 separate experiments. *P < 0.05, **P < 0.01, and ***P < 0.001.

Supplementary Figure 4. Changes in B cell activity in clinical HIV patients. The patients were divided into four groups: health individuals, HIV-infected patients, ART patients, and INR. A. Flow cytometric detection of BCR, TLR and co-stimulatory molecules (including CD40, CD80 and CD86). B. Western blot detection of BCR, TLR and co-stimulatory molecules (including CD40, CD80 and CD86). Data are represented as mean ± SD of 8 separate experiments. *P < 0.05, **P < 0.01, and ***P < 0.001.

## Data Availability

All data generated or analysed during this study are included in this article.

## References

[CR1] Bagheri Yazdi SM, Shahsavandi S, Fotouhi F, Tebianian M, Ebrahimi MM. Modulation of immune responses against HA1 influenza vaccine candidate by B-lymphocyte stimulator cytokine in mice. Iran J Allergy Asthma Immunol. 2022;21:207–14. 35490274 10.18502/ijaai.v21i2.9228

[CR2] Callahan LN, Roderiquez G, Mallinson M, Norcross MA. Analysis of HIV-induced autoantibodies to cryptic epitopes on human CD4. J Immunol. 1992;149:2194–202. 1381399

[CR3] Chams V, Jouault T, Fenouillet E, Gluckman JC, Klatzmann D. Detection of anti-CD4 autoantibodies in the Sera of HIV-infected patients using Recombinant soluble CD4 molecules. AIDS. 1988;2:353–61. 3146263 10.1097/00002030-198810000-00004

[CR4] Chams V, Idziorek T, Klatzmann D. Biological properties of anti-CD4 autoantibodies purified from HIV-infected patients. AIDS. 1991;5:565–9. 1677809

[CR5] Chriswell ME, Lefferts AR, Clay MR, Hsu AR, Seifert J, Feser ML, Rims C, Bloom MS, Bemis EA, Liu S, Maerz MD, Frank DN, Demoruelle MK, Deane KD, James EA, Buckner JH, Robinson WH, Holers VM, Kuhn KA. Clonal IgA and IgG autoantibodies from individuals at risk for rheumatoid arthritis identify an arthritogenic strain of Subdoligranulum. Sci Transl Med. 2022;14:eabn5166. 36288282 10.1126/scitranslmed.abn5166 PMC9804515

[CR6] Deng L, Hernandez N, Zhong L, Holcomb DD, Yan H, Virata ML, Tarafdar S, Xu Y, He Y, Struble E, Alter HJ, Zhang P. A conserved epitope III on hepatitis C virus E2 protein has alternate conformations facilitating cell binding or virus neutralization. Proc Natl Acad Sci U S A. 2021;118. 10.1073/pnas.2104242118 PMC8285954 34260404

[CR7] Di Modica M, Sfondrini L, Regondi V, Varchetta S, Oliviero B, Mariani G, Bianchi GV, Generali D, Balsari A, Triulzi T, Tagliabue E. Taxanes enhance trastuzumab-mediated ADCC on tumor cells through NKG2D-mediated NK cell recognition. Oncotarget. 2016;7:255–65. 26595802 10.18632/oncotarget.6353 PMC4807996

[CR8] Dickinson GS, Sun G, Bram RJ, Alugupalli KR. Efficient B cell responses to Borrelia hermsii infection depend on BAFF and BAFFR but not TACI. Infect Immun. 2014;82:453–9. 24218480 10.1128/IAI.01147-13 PMC3911873

[CR9] Evans LS, Lewis KE, DeMonte D, Bhandari JG, Garrett LB, Kuijper JL, Ardourel D, Wolfson MF, Debrot S, Mudri S, Kleist K, Griffin LL, Hebb L, Sanderson RJ, Wang N, Seaberg M, Chunyk AG, Yang J, Hong Y, Maria Z, Messenheimer DJ, Holland PM, Peng SL, Rixon MW, Dillon SR. Povetacicept, an enhanced dual APRIL/BAFF antagonist that modulates B lymphocytes and pathogenic autoantibodies for the treatment of lupus and other B Cell-Related autoimmune diseases. Arthritis Rheumatol. 2023;75:1187–202. 36705554 10.1002/art.42462

[CR10] Forthal DN, Finzi A. Antibody-dependent cellular cytotoxicity in HIV infection. AIDS. 2018;32:2439–51. 30234611 10.1097/QAD.0000000000002011 PMC6497078

[CR11] Han Y, Wu P, Wang Z, Zhang Z, Sun S, Liu J, Gong S, Gao P, Iwakuma T, Molina-Vila MA, Chen BP-C, Zhang Y, Ji T, Mo Q, Chen P, Hu J, Wang S, Zhou J, Lu H, Gao Q. Ubiquinol-cytochrome C reductase core protein II promotes tumorigenesis by facilitating p53 degradation. EBioMedicine. 2019;40. 10.1016/j.ebiom.2019.01.002 PMC6412871 30674441

[CR12] Huang S-F, Zhao G, Peng X-F, Ye W-C. The pathogenic role of long Non-coding RNA H19 in atherosclerosis via the miR-146a-5p/ANGPTL4 pathway. Front Cardiovasc Med. 2021;8:770163. 34820432 10.3389/fcvm.2021.770163 PMC8606739

[CR13] Hunt PW, Martin JN, Sinclair E, Bredt B, Hagos E, Lampiris H, Deeks SG. T cell activation is associated with lower CD4 + T cell gains in human immunodeficiency virus-infected patients with sustained viral suppression during antiretroviral therapy. J Infect Dis. 2003;187:1534–43. 12721933 10.1086/374786

[CR14] Ji T, Lang J, Ning B, Qi F, Wang H, Zhang Y, Zhao R, Yang X, Zhang L, Li W, Shi X, Qin Z, Zhao Y, Nie G. Enhanced natural killer cell immunotherapy by rationally assembling Fc fragments of antibodies onto tumor membranes. Adv Mater. 2019;31:e1804395. 30549110 10.1002/adma.201804395

[CR15] Khlaiphuengsin A, Chuaypen N, Sodsai P, Buranapraditkun S, Boonpiyathad T, Hirankarn N, Tangkijvanich P. Decreased of BAFF-R expression and B cells maturation in patients with hepatitis B virus-related hepatocellular carcinoma. World J Gastroenterol. 2020;26:2645–56. 32523317 10.3748/wjg.v26.i20.2645 PMC7265148

[CR16] Lane HC, Masur H, Edgar LC, Whalen G, Rook AH, Fauci AS. Abnormalities of B-cell activation and immunoregulation in patients with the acquired immunodeficiency syndrome. N Engl J Med. 1983;309:453–8. 6224088 10.1056/NEJM198308253090803

[CR17] Laopajon W, Cheyasawan P, Pata S, Takheaw N, Kasinrerk W. The ligation of CD4 molecules, expressed on monocytes by an anti-CD4 monoclonal antibody, inhibits T cell activation and monocyte mobility. Asian Pac J Allergy Immunol. 2023. 10.12932/AP-150123-1532 37302098

[CR18] Li M, Liu W, Bauch T, Graviss EA, Arduino RC, Kimata JT, Chen M, Wang J. Clearance of HIV infection by selective elimination of host cells capable of producing HIV. Nat Commun. 2020;11:4051. 32792548 10.1038/s41467-020-17753-w PMC7426846

[CR19] Li H, Boulougoura A, Endo Y, Tsokos GC. Abnormalities of T cells in systemic lupus erythematosus: new insights in pathogenesis and therapeutic strategies. J Autoimmun. 2022;132:102870. 35872102 10.1016/j.jaut.2022.102870

[CR20] Li M, Budai MM, Chen M, Wang J. Targeting HIV-1 reservoirs in T cell subsets. Front Immunol. 2023;14:1087923. 36742330 10.3389/fimmu.2023.1087923 PMC9895780

[CR21] Lin L, Couturier J, Yu X, Medina MA, Kozinetz CA, Lewis DE. Granzyme B secretion by human memory CD4 T cells is less strictly regulated compared to memory CD8 T cells. BMC Immunol. 2014;15:36. 25245659 10.1186/s12865-014-0036-1 PMC4195902

[CR22] Lu L-L, Xiao S-X, Lin Z-Y, Bai J-J, Li W, Song Z-Q, Zhou Y-H, Lu B, Wu W-Z. GPC3-IL7-CCL19-CAR-T primes immune microenvironment reconstitution for hepatocellular carcinoma therapy. Cell Biol Toxicol. 2023;39:3101–19. 37853185 10.1007/s10565-023-09821-w

[CR23] Luo Z, Li Z, Martin L, Wan Z, Meissner EG, Espinosa E, Wu H, Yu X, Fu P, Julia Westerink MA, Kilby JM, Wu J, Huang L, Heath SL, Li Z, Jiang W. Pathological role of Anti-CD4 antibodies in HIV-Infected Immunologic nonresponders receiving Virus-Suppressive antiretroviral therapy. J Infect Dis. 2017;216:82–91. 28498953 10.1093/infdis/jix223 PMC5853506

[CR24] Luo Y, Xiang K, Liu J, Song J, Feng J, Chen J, Dai Y, Hu Y, Zhuang H, Zhou Y. Inhibition of In Vitro Infection of Hepatitis B Virus by Human Breastmilk. Nutrients. 2022;14. 10.3390/nu14081561 PMC9031155 35458123

[CR25] Mackay F, Leung H. The role of the BAFF/APRIL system on T cell function. Semin Immunol. 2006;18:284–9. 16931039 10.1016/j.smim.2006.04.005

[CR26] Malle L, Patel RS, Martin-Fernandez M, Stewart OJ, Philippot Q, Buta S, Richardson A, Barcessat V, Taft J, Bastard P, Samuels J, Mircher C, Rebillat A-S, Maillebouis L, Vilaire-Meunier M, Tuballes K, Rosenberg BR, Trachtman R, Casanova J-L, Notarangelo LD, Gnjatic S, Bush D, Bogunovic D. Autoimmunity in down’s syndrome via cytokines, CD4 T cells and CD11c + B cells. Nature. 2023;615:305–14. 36813963 10.1038/s41586-023-05736-y PMC9945839

[CR27] Martin L, Idziorek T, Lehen A, Gluckman JC, Klatzmann D. Anti-CD4 autoantibodies in HIV-infected individuals: detection by two Immunoenzymatic techniques. AIDS. 1994;8:389–90. 8031521

[CR28] Mataramvura H, Bunders MJ, Duri K. Human immunodeficiency virus and antiretroviral therapy-mediated immune cell metabolic dysregulation in children born to HIV-infected women: potential clinical implications. Front Immunol. 2023;14:1182217. 37350953 10.3389/fimmu.2023.1182217 PMC10282157

[CR29] Menéndez-Arias L, Delgado R. Update and latest advances in antiretroviral therapy. Trends Pharmacol Sci. 2022;43:16–29. 34742581 10.1016/j.tips.2021.10.004

[CR30] Mielke D, Bandawe G, Zheng J, Jones J, Abrahams M-R, Bekker V, Ochsenbauer C, Garrett N, Karim A, Moore S, Morris PL, Montefiori L, Anthony D, Ferrari C, G., Williamson C. ADCC-mediating non-neutralizing antibodies can exert immune pressure in early HIV-1 infection. PLoS Pathog. 2021;17:e1010046. 34788337 10.1371/journal.ppat.1010046 PMC8598021

[CR31] Müller C, Kukel S, Bauer R. Relationship of antibodies against CD4 + T cells in HIV-infected patients to markers of activation and progression: autoantibodies are closely associated with CD4 cell depletion. Immunology. 1993;79:248–54. 8102120 PMC1421877

[CR32] Nahmad AD, Raviv Y, Horovitz-Fried M, Sofer I, Akriv T, Nataf D, Dotan I, Carmi Y, Burstein D, Wine Y, Benhar I, Barzel A. Engineered B cells expressing an anti-HIV antibody enable memory retention, isotype switching and clonal expansion. Nat Commun. 2020;11:5851. 33203857 10.1038/s41467-020-19649-1 PMC7673991

[CR33] Nilssen DE, Øktedalen O, Brandtzaeg P. Intestinal B cell hyperactivity in AIDS is controlled by highly active antiretroviral therapy. Gut. 2004;53:487–93. 15016741 10.1136/gut.2003.027854 PMC1774010

[CR34] Robbins GK, Spritzler JG, Chan ES, Asmuth DM, Gandhi RT, Rodriguez BA, Skowron G, Skolnik PR, Shafer RW, Pollard RB. Incomplete reconstitution of T cell subsets on combination antiretroviral therapy in the AIDS clinical trials group protocol 384. Clin Infect Dis. 2009;48:350–61. 19123865 10.1086/595888 PMC2676920

[CR35] Roth A, Glaesener S, Schütz K, Meyer-Bahlburg A. Reduced number of transitional and Naive B cells in addition to decreased BAFF levels in response to the T cell independent immunogen Pneumovax^®^23. PLoS ONE. 2016;11:e0152215. 27031098 10.1371/journal.pone.0152215 PMC4816312

[CR36] Sakai J, Akkoyunlu M. The role of BAFF system molecules in host response to pathogens. Clin Microbiol Rev. 2017;30. 10.1128/CMR.00046-17 PMC5608883 28855265

[CR37] Sekigawa I, Groopmen JE, Allan JD, Ikeuchi K, Biberfield G, Takatsuki K, Byrn RA. Characterization of autoantibodies to the CD4 molecule in human immunodeficiency virus infection. Clin Immunol Immunopathol. 1991;58:145–53. 1983967 10.1016/0090-1229(91)90156-5

[CR38] Sheikh V, Dersimonian R, Richterman AG, Porter BO, Natarajan V, Burbelo PD, Rupert A, Santich BH, Kardava L, Mican JM, Moir S, Sereti I. Graves’ disease as immune reconstitution disease in HIV-positive patients is associated with Naive and primary thymic emigrant CD4(+) T-cell recovery. AIDS. 2014;28:31–9. 23939238 10.1097/QAD.0000000000000006

[CR39] Shirai A, Cosentino M, Leitman-Klinman SF, Klinman DM. Human immunodeficiency virus infection induces both polyclonal and virus-specific B cell activation. J Clin Invest. 1992;89:561–6. 1737846 10.1172/JCI115621 PMC442888

[CR40] Siloşi I, Siloşi CA, Boldeanu MV, Cojocaru M, Biciuşcă V, Avrămescu CS, Cojocaru IM, Bogdan M, FolcuŢi RM. The role of autoantibodies in health and disease. Rom J Morphol Embryol. 2016;57:633–8. 27833954

[CR41] Singh B, Sharan R, Ravichandran G, Escobedo R, Shivanna V, Dick EJ, Hall-Ursone S, Arora G, Alvarez X, Singh DK, Kaushal D, Mehra S. Indoleamine-2,3-dioxygenase Inhibition improves immunity and is safe for concurrent use with cART during mtb/siv coinfection. JCI Insight. 2024;9. 10.1172/jci.insight.179317 PMC11383603 39114981

[CR42] Smulski CR, Eibel H. BAFF and BAFF-Receptor in B cell selection and survival. Front Immunol. 2018;9:2285. 30349534 10.3389/fimmu.2018.02285 PMC6186824

[CR43] Strunz B, Hengst J, Deterding K, Manns MP, Cornberg M, Ljunggren H-G, Wedemeyer H, Björkström NK. Chronic hepatitis C virus infection irreversibly impacts human natural killer cell repertoire diversity. Nat Commun. 2018;9:2275. 29891939 10.1038/s41467-018-04685-9 PMC5995831

[CR44] Swanstrom AE, Immonen TT, Oswald K, Pyle C, Thomas JA, Bosche WJ, Silipino L, Hull M, Newman L, Coalter V, Wiles A, Wiles R, Kiser J, Morcock DR, Shoemaker R, Fast R, Breed MW, Kramer J, Donohue D, Malys T, Fennessey CM, Trubey CM, Deleage C, Estes JD, Lifson JD, Keele BF, Del Prete GQ. Antibody-mediated depletion of viral reservoirs is limited in SIV-infected macaques treated early with antiretroviral therapy. J Clin Invest. 2021;131. 10.1172/JCI142421 PMC7954603 33465055

[CR45] Towriss M, MacVicar B, Ciernia AV. Modelling microglial innate immune memory in vitro: Understanding the role of aerobic Glycolysis in innate immune memory. Int J Mol Sci. 2023;24. 10.3390/ijms24108967 PMC10219556 37240311

[CR46] Vos WAJW, Navas A, Meeder EMG, Blaauw MJT, Groenendijk AL, van Eekeren LE, Otten T, Vadaq N, Matzaraki V, van Cranenbroek B, Brinkman K, van Lunzen J, Joosten LAB, Netea MG, Blok WL, van der Ven AJAM, Koenen HJPM, Stalenhoef JE. HIV immunological non-responders are characterized by extensive Immunosenescence and impaired lymphocyte cytokine production capacity. Front Immunol. 2024;15:1350065. 38779686 10.3389/fimmu.2024.1350065 PMC11109418

[CR47] Williams KL, Cortez V, Dingens AS, Gach JS, Rainwater S, Weis JF, Chen X, Spearman P, Forthal DN, Overbaugh J. HIV-specific CD4-induced antibodies mediate broad and potent Antibody-dependent cellular cytotoxicity activity and are commonly detected in plasma from HIV-infected humans. EBioMedicine. 2015;2:1464–77. 26629541 10.1016/j.ebiom.2015.09.001 PMC4634620

[CR48] Wu H, Su S, Wu Y, Wu Y, Zhang Z, Chen Q. Nanoparticle-facilitated delivery of BAFF-R SiRNA for B cell intervention and rheumatoid arthritis therapy. Int Immunopharmacol. 2020;88:106933. 32866781 10.1016/j.intimp.2020.106933

[CR49] Xiao Q, Yan L, Han J, Yang S, Tang Y, Li Q, Lao X, Chen Z, Xiao J, Zhao H, Yu F, Zhang F. Metabolism-dependent ferroptosis promotes mitochondrial dysfunction and inflammation in CD4 + T lymphocytes in HIV-infected immune non-responders. EBioMedicine. 2022;86:104382. 36462403 10.1016/j.ebiom.2022.104382 PMC9718960

[CR50] Xu J, Liu O, Wang D, Wang F, Zhang D, Jin W, Cain A, Bynum A, Liu N, Han Y, Chen W. In vivo generation of ssa/ro Antigen-Specific regulatory T cells improves experimental sjögren’s syndrome in mice. Arthritis Rheumatol. 2022;74:1699–705. 35606923 10.1002/art.42244 PMC9811988

[CR51] Yaffe ZA, Naiman NE, Slyker J, Wines BD, Richardson BA, Hogarth PM, Bosire R, Farquhar C, Ngacha DM, Nduati R, John-Stewart G, Overbaugh J. Improved HIV-positive infant survival is correlated with high levels of HIV-specific ADCC activity in multiple cohorts. Cell Rep Med. 2021;2:100254. 33948582 10.1016/j.xcrm.2021.100254 PMC8080236

[CR52] Yang X, Su B, Zhang X, Liu Y, Wu H, Zhang T. Incomplete immune reconstitution in HIV/AIDS patients on antiretroviral therapy: challenges of immunological non-responders. J Leukoc Biol. 2020;107:597–612. 31965635 10.1002/JLB.4MR1019-189R PMC7187275

[CR53] Yao Y, Ma J-F, Chang C, Xu T, Gao C-Y, Gershwin ME, Lian Z-X. Immunobiology of T cells in sjögren’s syndrome. Clin Rev Allergy Immunol. 2021;60:111–31. 32390096 10.1007/s12016-020-08793-7

[CR54] Ye W, Chen R, Chen X, Huang B, Lin R, Xie X, Chen J, Jiang J, Deng Y, Wen J. AhR regulates the expression of human cytochrome P450 1A1 (CYP1A1) by recruiting Sp1. FEBS J. 2019a;286:4215–31. 31199573 10.1111/febs.14956

[CR55] Ye W, Lin R, Chen X, Chen J, Chen R, Xie X, Deng Y, Wen J. T-2 toxin upregulates the expression of human cytochrome P450 1A1 (CYP1A1) by enhancing NRF1 and Sp1 interaction. Toxicol Lett. 2019b;315:77–86. 31470059 10.1016/j.toxlet.2019.08.021

[CR56] Zandman-Goddard G, Shoenfeld Y. HIV and autoimmunity. Autoimmun Rev. 2002;1:329–37. 12848988 10.1016/s1568-9972(02)00086-1

[CR57] Zhao J, Schank M, Wang L, Li Z, Nguyen LN, Dang X, Cao D, Khanal S, Nguyen LNT, Thakuri BKC, Ogbu SC, Lu Z, Wu XY, Morrison ZD, Gazzar ME, Liu Y, Zhang J, Ning S, Moorman JP, Yao ZQ. Mitochondrial functions are compromised in CD4 T cells from ART-Controlled PLHIV. Front Immunol. 2021;12:658420. 34017335 10.3389/fimmu.2021.658420 PMC8129510

