# Supplementary figures and images for: Effect of anti-CD4 mAb induced by inhibiting B cell disorder on immune reconstruction of HIV-infected immunological non-responders
Source: Mol Med. 2025 Jun 20;31:244. doi: 10.1186/s10020-025-01286-3 (PMC12180279; doi:10.1186/s10020-025-01286-3)

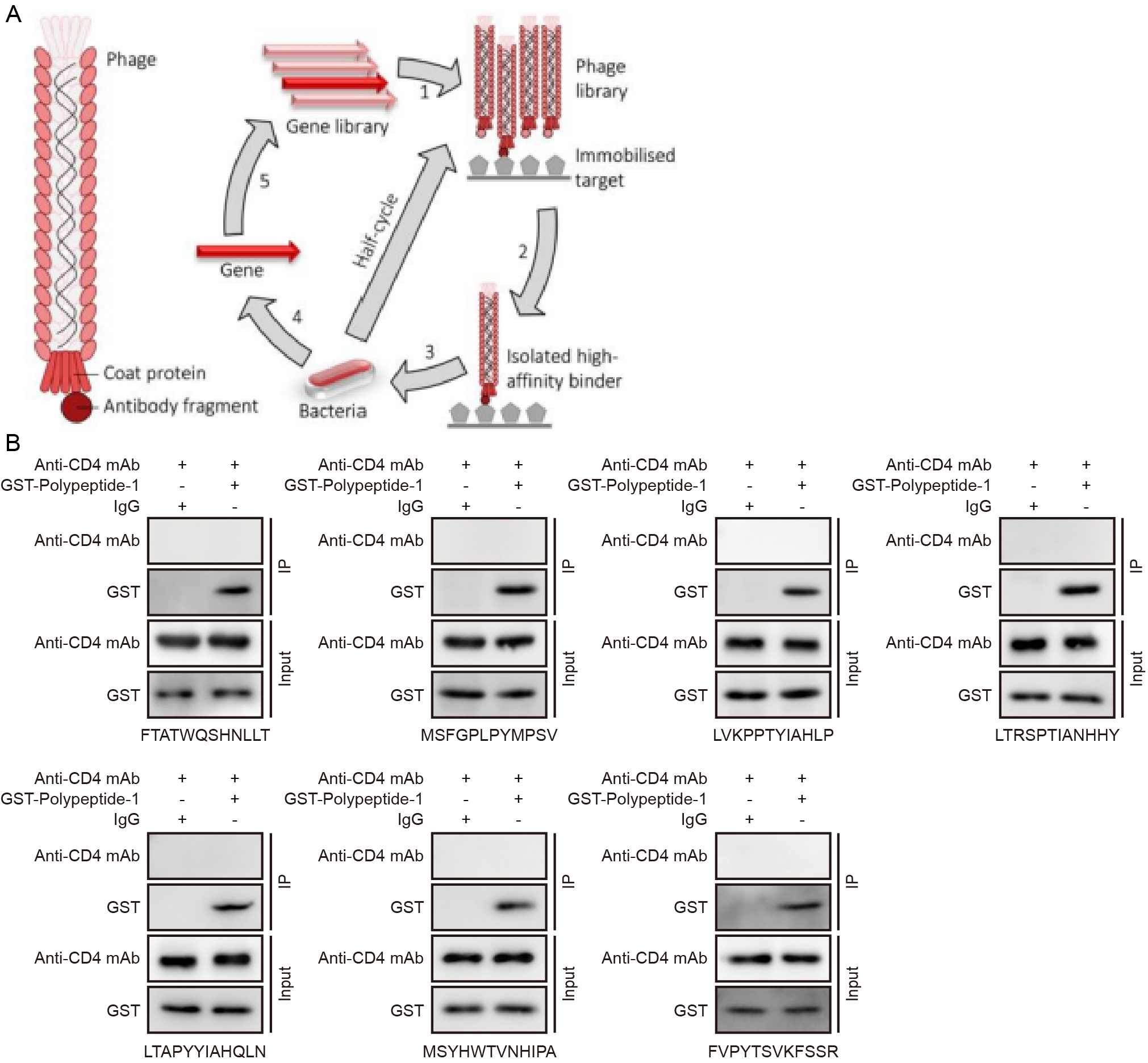

Supplement: Supplementary file 1 — Supplementary Figure 1. Detection of binding of CD4 autoantibodies to target epitopes. There were 7 polypeptides: 1) FTATWQSHNLLT; 2) MSFGPLPYMPSV; 3) LVKPPTYIAHLP; 4) LTRSPTIANHHY; 5) LTAPYVIAHQLN; 6) MSYHWTVNHIPA; 7) FVPYTSVKFSSR. A. Phage Random Peptide Library Screening. B. GST-Pulldown detection of anti-CD4 mAb binding to CD4 Peptide. [file 10020_2025_1286_MOESM1_ESM.tif]

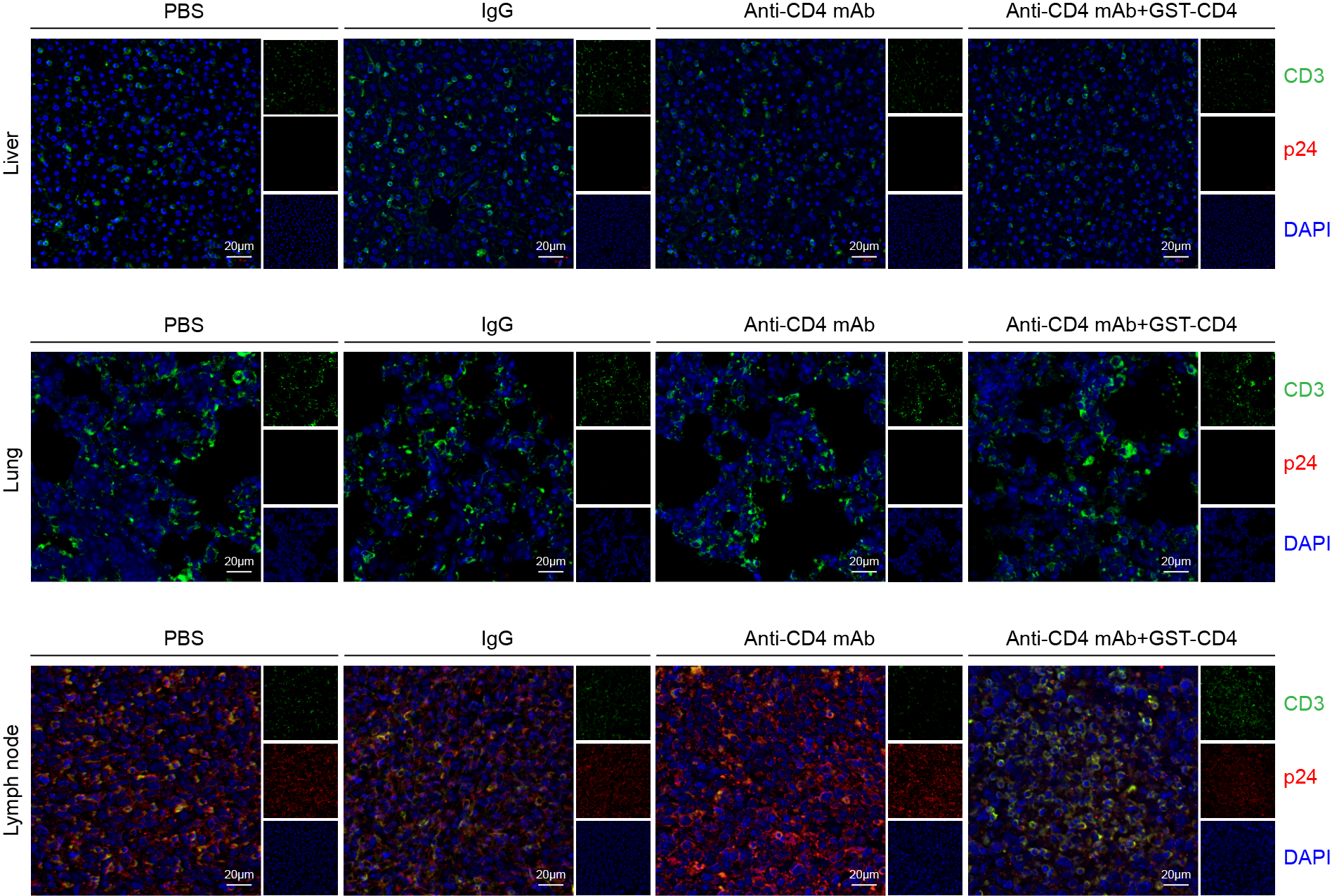

Supplement: Supplementary file 2 — Supplementary Figure 2. Effects of anti-CD4 autoantibodies on CD4+ cells in liver, lung, and lymph nodes in a humanized mouse model. A. Immunofluorescence detection of CD4+ T cells in mouse liver, lung, and lymph node tissues. [file 10020_2025_1286_MOESM2_ESM.tif]

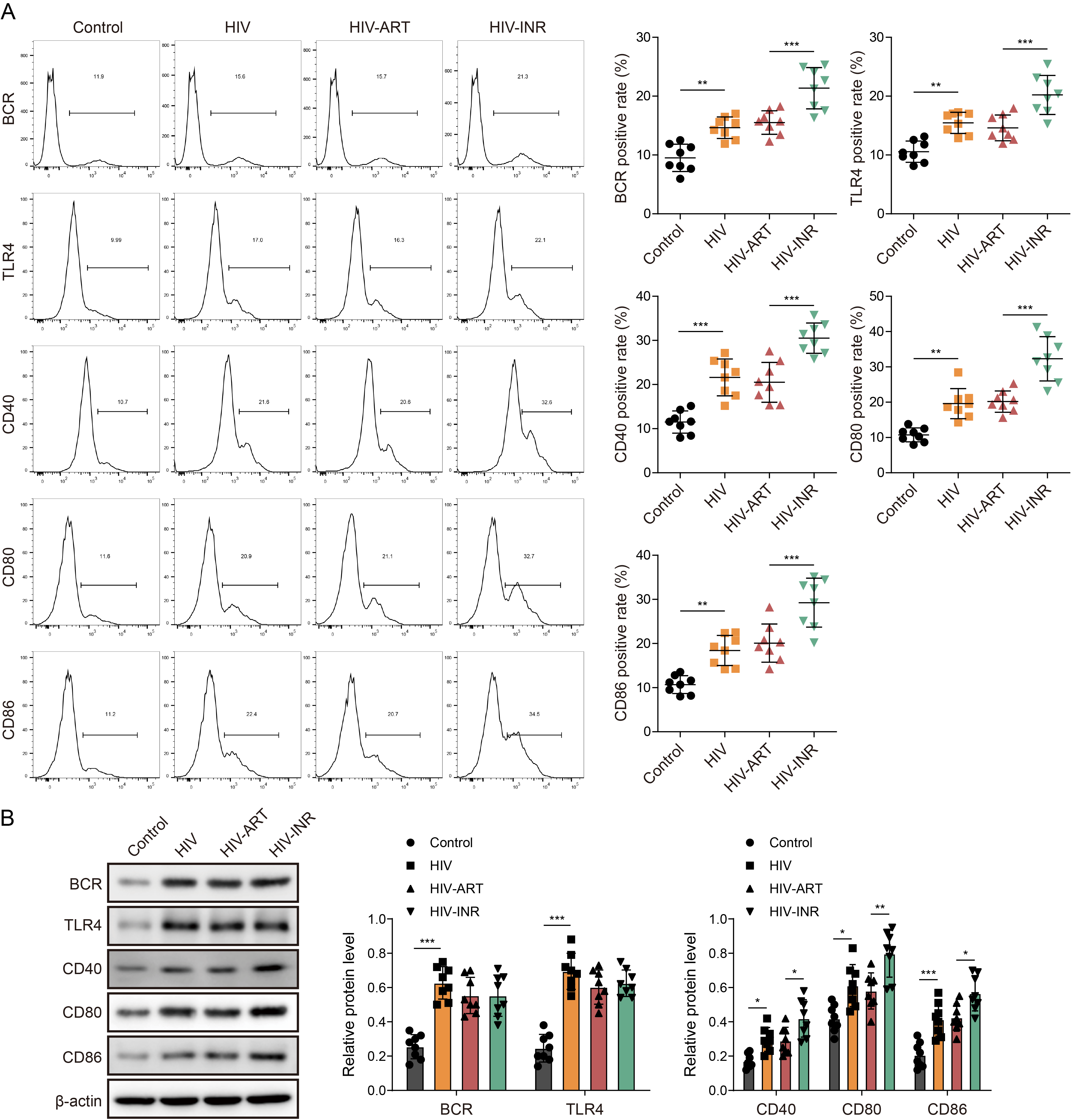

Supplement: Supplementary file 3 — Supplementary Figure 3. Changes in B cell activity in clinical HIV patients. The patients were divided into four groups: health individuals, HIV-infected patients, ART patients, and INR. A. Flow cytometric detection of BCR, TLR and co-stimulatory molecules (including CD40, CD80 and CD86). B. Western blot detection of BCR, TLR and co-stimulatory molecules (including CD40, CD80 and CD86). Data are represented as mean ± SD of 8 separate experiments. *P < 0.05, **P < 0.01, and ***P < 0.001. [file 10020_2025_1286_MOESM3_ESM.tif]

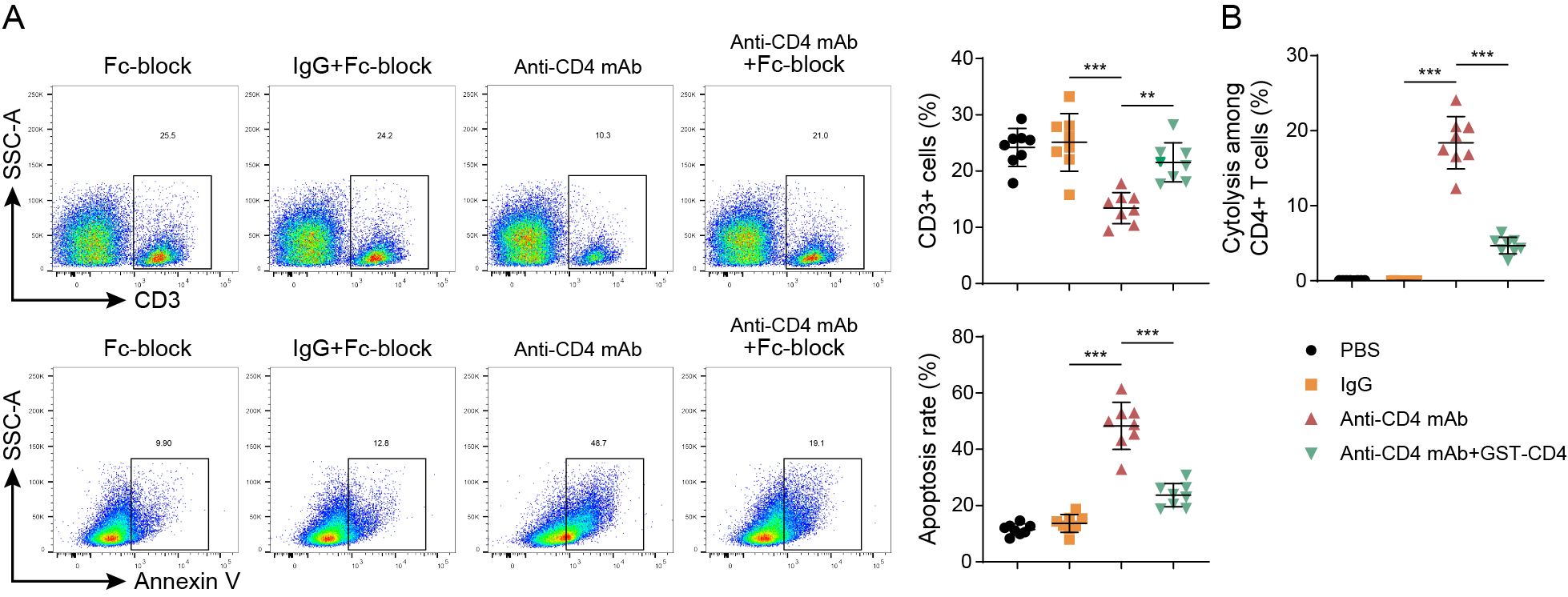

Supplement: Supplementary file 4 — Supplementary Figure 4. Changes in B cell activity in clinical HIV patients. The patients were divided into four groups: health individuals, HIV-infected patients, ART patients, and INR. A. Flow cytometric detection of BCR, TLR and co-stimulatory molecules (including CD40, CD80 and CD86). B. Western blot detection of BCR, TLR and co-stimulatory molecules (including CD40, CD80 and CD86). Data are represented as mean ± SD of 8 separate experiments. *P < 0.05, **P < 0.01, and ***P < 0.001. [file 10020_2025_1286_MOESM4_ESM.tif]
